# Synthesis and Chemiluminescent Properties of Amino-Acylated luminol Derivatives Bearing Phosphonium Cations

**DOI:** 10.3390/molecules24213957

**Published:** 2019-10-31

**Authors:** Anna Pantelia, Ira Daskalaki, M. Consuelo Cuquerella, Georgios Rotas, Miguel A. Miranda, Georgios C. Vougioukalakis

**Affiliations:** 1 Laboratory of Organic Chemistry, Department of Chemistry, National and Kapodistrian University of Athens, Panepistimiopolis, 15771 Athens, Greece; annapantelia@gmail.com (A.P.); iradask@hotmail.com (I.D.); rotasgiorgos@hotmail.com (G.R.); 2 Instituto de Tecnología Química (UPV-CSIC), Universitat Politècnica de València, 46022 València, Spain; mcuquere@itq.upv.es

**Keywords:** phthalhydrazide, luminol, chemiluminescence, peroxide, phosphonium, mitochondria

## Abstract

The monitoring of reactive oxygen species in living cells provides valuable information on cell function and performance. Lately, the development of chemiluminescence-based reactive oxygen species monitoring has gained increased attention due to the advantages posed by chemiluminescence, including its rapid measurement and high sensitivity. In this respect, specific organelle-targeting trackers with strong chemiluminescence performance are of high importance. We herein report the synthesis and chemiluminescence properties of eight novel phosphonium-functionalized amino-acylated luminol and isoluminol derivatives, designed as mitochondriotropic chemiluminescence reactive oxygen species trackers. Three different phosphonium cationic moieties were employed (phenyl, *p*-tolyl, and cyclohexyl), as well as two alkanoyl chains (hexanoyl and undecanoyl) as bridges/linkers. Synthesis is accomplished via the acylation of the corresponding phthalimides, as phthalhydrazide precursors, followed by hydrazinolysis. This method was chosen because the direct acylation of (iso)luminol was discouraging. The new derivatives’ chemiluminescence was evaluated and compared with that of the parent molecules. A relatively poor chemiluminescence performance was observed for all derivatives, with the isoluminol-based ones being the poorest. This result is mainly attributed to the low yield of the fluorescence species formation during the chemiluminescence oxidation reaction.

## 1. Introduction

Chemiluminescence (CL), the emission of light derived from a chemical reaction, is usually the outcome of a substrate’s redox reaction towards the formation of an excited species, which emits light upon deactivation [1]. This chemically-induced light generation is of high importance, for both detection and analytical purposes, finding applications in analytical chemistry, clinical diagnostics, forensics, etc. [2,3,4]. High sensitivity, linear response, and fast measurement are among the main advantages of CL-based analyses. In parallel, the growing evidence on the importance of certain highly-reactive oxidants, known as reactive oxygen and nitrogen species (ROS and RNS, respectively), in cell signaling, homeostasis, and metabolism [5,6,7,8] has necessitated the development of methods for detecting the intracellular levels of ROS/RNS [9,10]. In this regard, mitochondria have been identified as the primary ROS-producing organelles [11,12,13]. The existence of ROS/RNS, both ideal oxidants in CL reactions, has led to the use of chemiluminescent ROS-detecting probes, capitalizing on the advantages of CL-based analyses [14,15,16,17,18].

5-Amino-2,3-dihydrophthalazine-1,4-dione is probably the most notorious and widely-used chemiluminescent reagent [19,20,21]. It was synthesized for the first time at the beginning of the 20th century [22], but its outstanding CL properties were discovered 26 years later [23]. The nickname “Luminol” was given to this bicyclic compound, due to its intriguing CL properties [24], while fame came a little later, when it was first reported as an efficient blood tracker in forensics [25]. Luminol’s strong chemiluminescence is triggered upon oxidation from peroxide in the presence of a catalyst (peroxidases, Fe^3+^, HOCl), yielding the excited 3-aminophthalate anion, which has been identified as the light-emitting species. Although its laboratory use is widespread, it has been only recently employed as in vivo CL tracker of neutrophil anti-microbial activity, either unmodified [26,27], or as a functional biodegradable material [28]. In this respect, functionalization of luminol with targeting moieties is expected to result in novel organelle-specific molecular trackers.

Our present work is part of an ongoing collaborative project on the development of novel, mitochondriotropic chemiluminescent probes for ROS detection. In this regard, we opt for the synthesis of tailor-designed luminol and isoluminol (the 6-amino isomer of luminol) derivatives, covalently linked with phosphonium cations as mitochondriotropic moieties [29,30,31]. Herein, we report on the synthesis and chemiluminescent properties of amino-acylated luminol and isoluminol derivatives bearing variable phosphonium cations (triphenyl, tris(4-tolyl) or tricyclohexyl) and chain lengths (hexyl, undecyl). Amino-acylation of (iso)luminol was chosen due to the seemingly ease of synthesis. Despite luminol’s high repute, chemically modified derivatives are rather limited. Simple acylated luminol derivatives have been synthesized and their chemiluminescence efficiencies have been evaluated more than 50 years ago [32,33,34]. Their synthesis was reportedly performed via the direct acylation of luminol with acyl chlorides, while their CL efficiencies appeared to be much lower to those of luminol, indicating that chemical functionalization can substantially alter the CL properties of the parent molecule. On the other hand, highly-efficient (electro)chemiluminescent luminol-Ru(bpy)_3_ donor-acceptor dyads [35,36] have been lately prepared from the direct acylation of luminol, while acylation has very recently been also achieved using cyclic anhydrides [37]. Thus, our goal was two-fold, namely: a) the development of an efficient synthetic procedure for a series of luminol and isoluminol amino-acylated phosphonium derivatives; and b) the evaluation of the chemiluminescence properties of these derivatives, in order to assess their potential for in vivo CL performance. Along these lines, a re-evaluation of the CL properties of acyl-(iso)luminol derivatives, studied by modern spectroscopic techniques, is presented herein.

## 2. Results

### 2.1. Synthesis of the Target Compounds

**Scheme 1:**
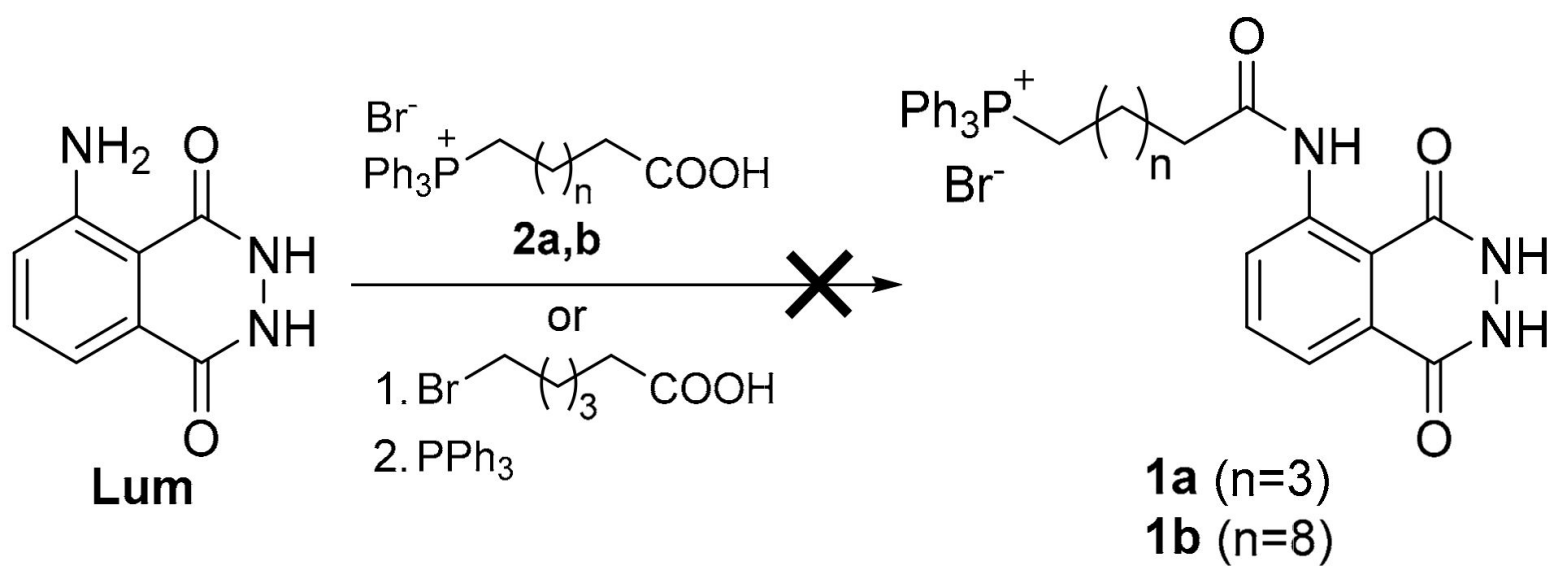
Attempted direct acylation of luminol.

The synthesis of amino-acylated luminol derivatives **1** was initially approached through the direct acylation of luminol (**Lum**) with a phosphonium-carboxylic acid derivative, albeit without success (Scheme 1). A variety of methods were employed, involving the reaction of luminol with phosphonium alkanoic acids **2a** or **2b**, (prepared from the reaction of 6-bromo-hexanoic or undecanoic acid with triphenylphosphine) [38] through acid chloride [32], or using coupling agents, yielding, in both cases, inseparable mixtures from which the desired product **1** could not be isolated in sufficient purity and yield. Other approaches, involving the use of NHS-activated esters, anhydride (prepared from DCC-mediated condensation of **2** [39]), or mixed anhydride (prepared in situ from **2** using ethyl chloroformate [40]), yielded again either inseparable mixtures, or no reaction. Preparation of **1** was also attempted using bromoalkanoic acids, followed by their reaction with phosphine, again without success.

**Scheme 2:**
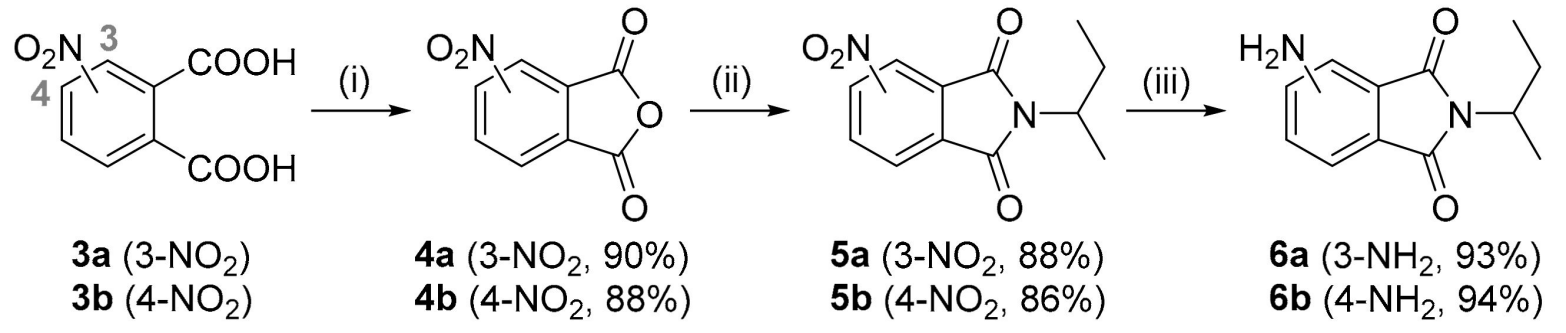
Synthesis of aminophthalimides **6a**,**b**. Reagents and conditions: (i) Ac_2_O, Δ; (ii) *sec*-BuNH_2_, AcOH, Δ; (iii) H_2_, Pd/C, MeOH.

The above disappointing results led to another synthetic approach. Both the acidity of luminol’s hydrazide protons and the weak nucleophilicity of its amino group were identified as potential source of byproducts (through 2-*N*-, 3-*N*-, or *O*-acylation). Therefore, amino-acylation was attempted on protected luminol derivatives. Phthalimides have been used as protected phthalhydrazides in the preparation of amino-alkylated isoluminol derivatives [41,42,43]. In this respect, amino-phthalimides **6a**,**b** (Scheme 2) were prepared from the respective nitrophthalic acids **3a**,**b** in a three-step reaction sequence involving consecutive condensation reactions towards anhydrides **4** and then phthalimides **5**, and finally reduction of the nitro group [41,44]. Phthalimides **6a**,**b** show good solubility in common organic solvents and thus can be handled easier, as compared to the respective phthalhydrazides. Additionally, their easy and scalable preparation (no column chromatography needed) render them valuable intermediates in the synthesis of phthalhydrazide derivatives.

**Scheme 3:**
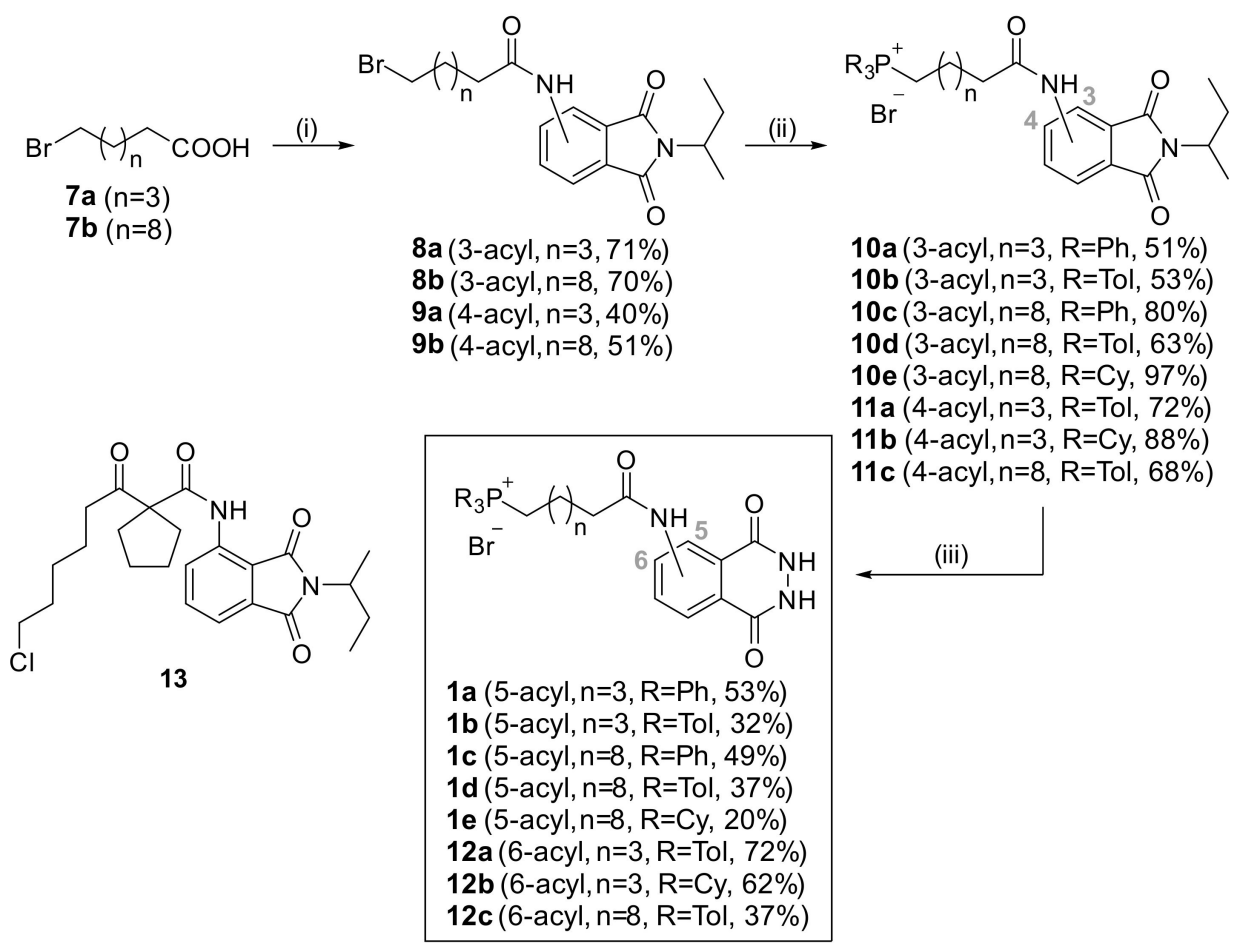
Synthesis of phosphonium bearing phthalhydrazides. Reagents and conditions: (i) (a) (COCl)_2_, (b) **6a** or **6b**, Py, DCM; (ii) PR_3_, MeCN, Δ; (iii) NH_2_NH_2_.H_2_O, EtOH, Δ. Yields are non-optimized.

Acylation of phthalimides **6a**,**b** proceeds smoothly with bromoalkyl carboxylic acids **7** via acyl chloride, furnishing the acylated phthalimides in moderate yields (Scheme 3). It is worth noting that room temperature has to be maintained throughout the reaction (even during the evaporation of oxalyl chloride), since halogen exchange occurs to some extent, towards the chloride, while more complex byproducts (e.g., **13** [45]) are isolated on prolonged heating. On the other hand, no reaction occurred when the acylation was attempted with the aid of coupling reagents (N-(3-dimethylaminopropyl)-N′-ethylcarbodiimide hydrochloride (EDC), N,N′-Dicyclohexylcarbodiimide (DCC) or vanillin, 4-dimethylaminopyridine (DMAP)). This result was in stark contrast to that of the similar coupling reaction of luminol mentioned above, where complex mixtures were formed. This is a clear indication that the hydrazide group is the source of by-product(s) formation. Next, introduction of the appropriate phosphine was performed in refluxing acetonitrile, yielding the corresponding phosphonium cations in moderate to good yields. Tricyclohexylphosphonium derivative **11b** was isolated as mixture with tricyclohexylphosphinoxide and was used in the next step as such. Phosphonium **1a** has been also prepared from the direct acylation of phthalimide **6a** with phosphonium carboxylic acid **2a** (via acyl chloride) in 61% yield, rendering this procedure a good alternative. Finally, hydrazinolysis of the phthalimides afforded the desired phthalhydrazides **1** and **12** in moderate (non-optimized) yields. Prolonged reaction times and high temperatures during hydrazinolysis have to be avoided in order to bypass amide bond cleavage.

**Figure 1:**
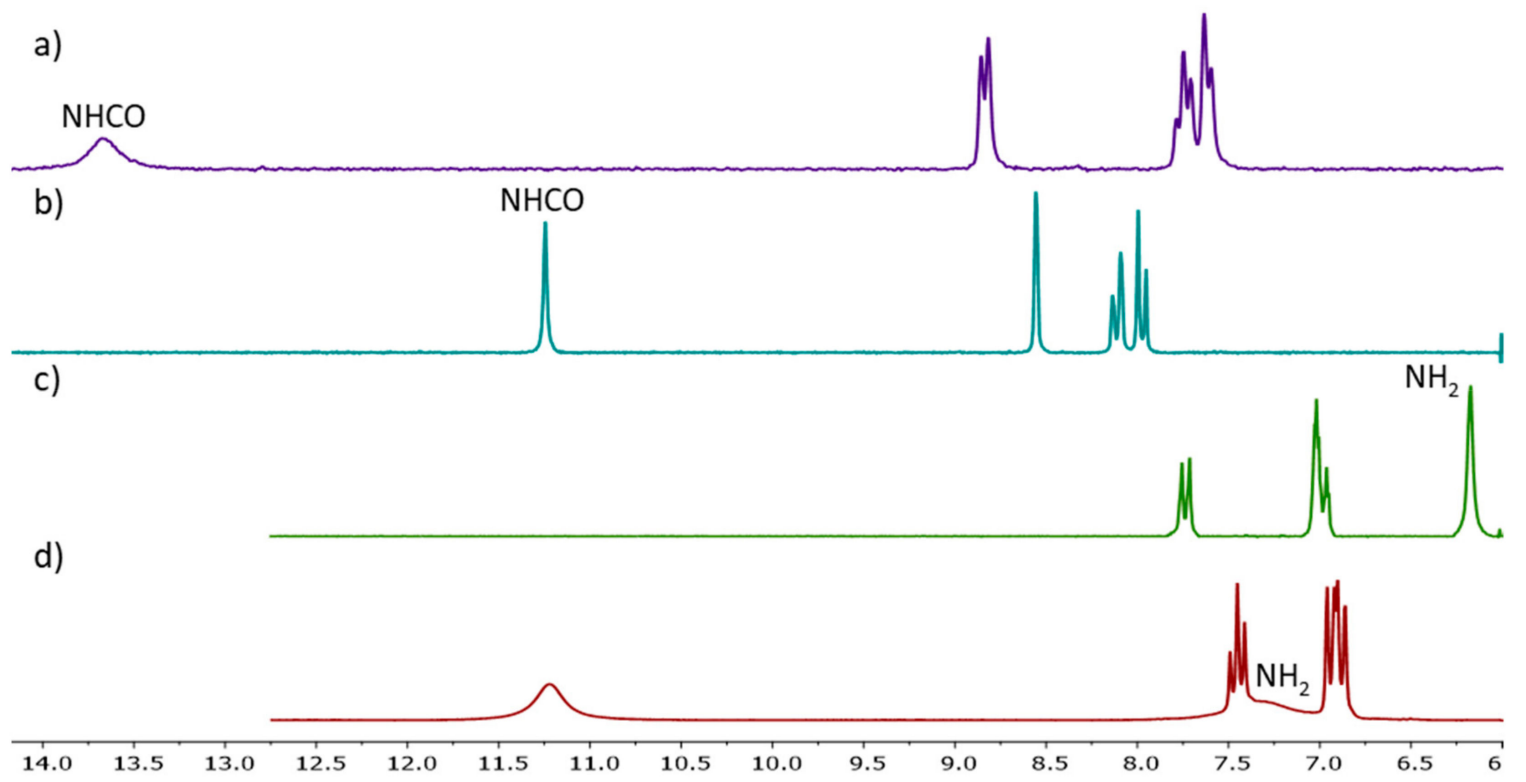
^1^H-NMR (200 MHz, DMSO-*d*_6_) spectra of: (**a**) **1e**; (**b**) **12b**; (**c**) isoluminol; and (**d**) luminol (aromatic region). The signals attributed to 5-*N*-amide (**a**,**b**) and amine (**c**,**d**) protons are indicated.

The desired products can be thus isolated in a repeatable manner and employing the usual purification procedures. Acylation of luminol and isoluminol is evident in the ^1^H-NMR spectra of the derivatives, where characteristic patterns appear, as shown in Figure 1. All signals are shifted downfield, as compared to their parent compounds, while the newly-obtained amide NH protons appear quite deshielded. The hydrazide proton signals are not always evident, usually appearing as very broad peaks of variable chemical shift (12–8 ppm), quite sensitive to moisture, solvent traces, and sample concentrations.

### 2.2. Chemiluminescence Studies

**Figure 2:**
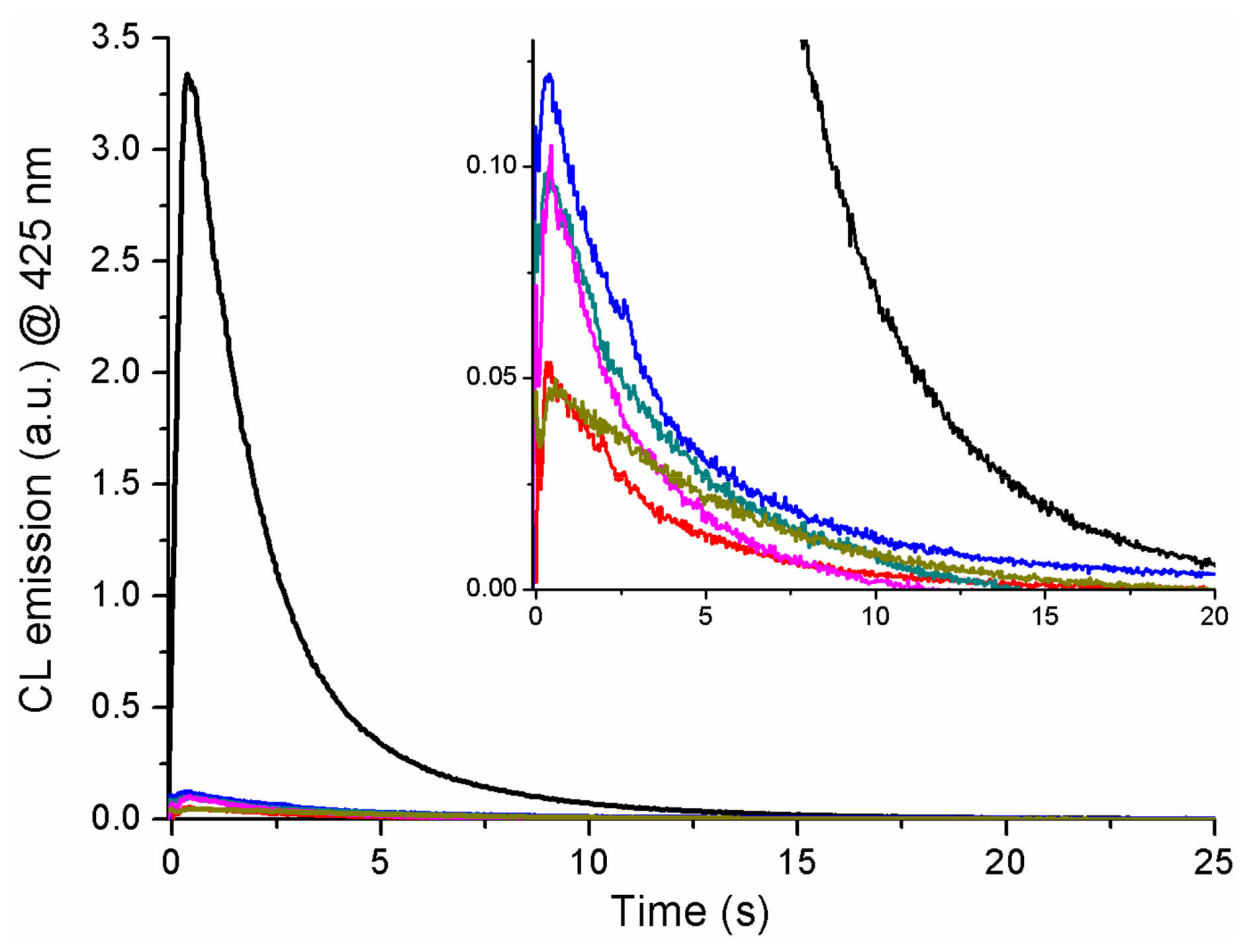
Results of a typical experiment to determine the chemiluminescence of the amino-acylated derivatives **1a** (blue), **1b** (green), **1c** (red), **1d** (magenta), **1e** (olive), and luminol (black) in aqueous basic solutions. Inset: magnification.

In order to investigate the CL properties of the new luminol-phosphonium derivatives, a protocol was established. Briefly, (iso)luminols were dissolved in aqueous basic solutions, giving a final concentration of 7.5 μM. Then, each sample was introduced in a quartz cuvette and the CL was triggered by subsequent addition of H_2_O_2_ and K_3_[Fe(CN)_6_] while vigorously stirring. Monitoring of the process was performed using a fluorometer with its own lamp switched off, running in the time-based mode. The CL displayed by the amino-acylated derivatives **1** and **12**, under these experimental conditions, together with that of the parent compound luminol, are shown in Figure 2 and Table 1.

As a general finding, acylation diminishes almost quantitatively luminol’s CL, irrespective of the mitotropic moieties incorporated to the structure, or the length of the chain employed to link them to the amino acylated luminols. Amino-acylated isoluminols (compounds **12**) showed even a more dramatic effect, as CL was practically undetectable. Chemiluminescence quantum yields (Φ_CL_) were determined by comparison with luminol, taking its previously established absolute Φ^0^_CL_ as 0.012 [46]. To do so, the total area under the curve for each compound was measured 10 times, in separate experiments, and the average value was used to calculate the relative Φ_CL_ as Φ_CL_ = A/A_L_ x Φ^0^_CL_, where A corresponds to the average area for each compound and A_L_ the value obtained in the case of the reference compound luminol. The results are listed in Table 1.

**Scheme 4:**
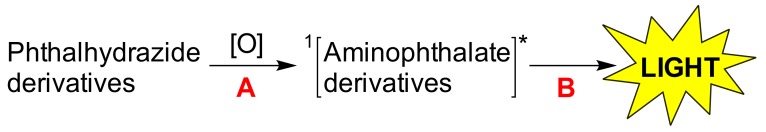
Steps involved in phthalhydrazide derivatives’ chemiluminescence.

**Figure 3:**
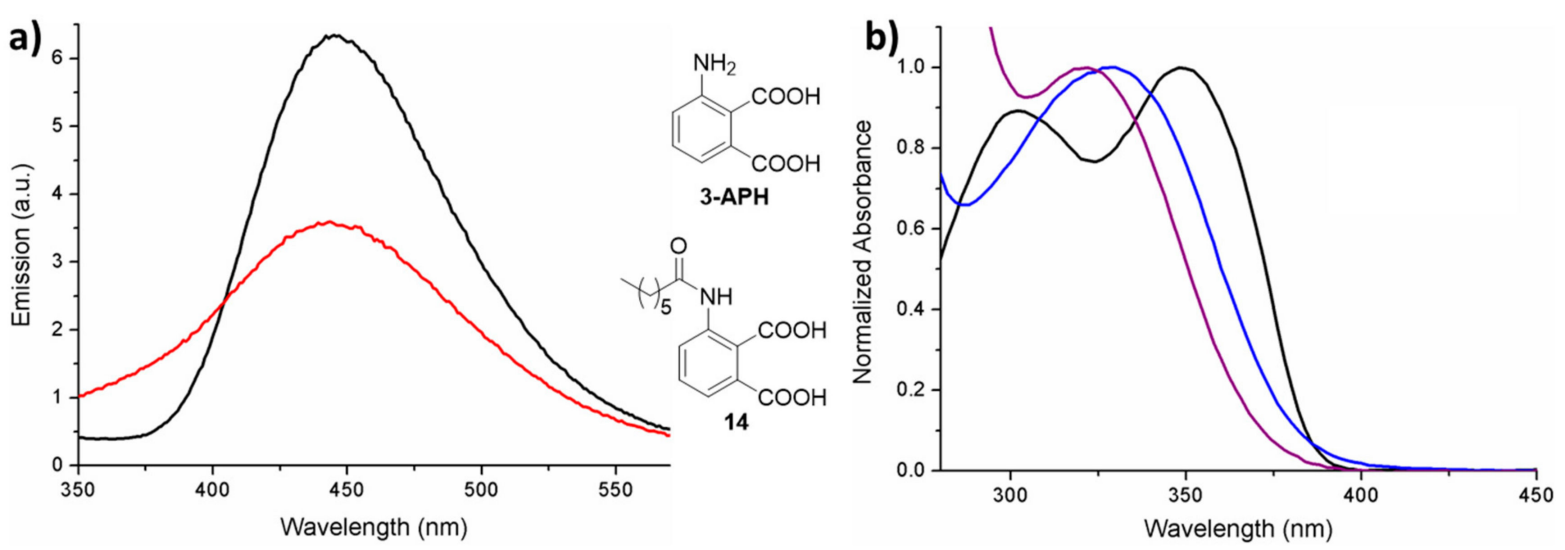
(**a**) Fluorescence spectra of **3APH** (black) and **14** (red) (λ_exc_ = 303 nm, matching absorbance) in aqueous basic solutions. (**b**) Normalized UV–vis spectra of luminol (black), **1a** (blue) and **12a** (violet) in aqueous basic solutions.

It is widely accepted that the oxidation of luminol leads to the formation of 3-aminophthalate (**3AP**) in its excited singlet state (Scheme 4, step A). In part, this excited species (^1^3AP*) relaxes to its ground state through the emission of light at 425 nm (Scheme 4, step B), thus producing the observed chemiluminescence in the global process. Taking this into account, the dramatic decrease of CL upon acylation could originate by a diminution in the yield of the oxidation and/or the emission steps. To ascertain which is the key step affected, the fluorescence of 3-heptanamidophthalic acid **14** was measured as reference compound (closely related to the emitting species in the CL of **1**, prepared in two steps from 3-nitrophthalic acid) and compared to that of **3APH** (Figure 3a) [47]. To this end, two alkaline isoabsorptive solutions of **3APH** and **14** were excited at the same wavelength (λ_exc_ = 303 nm) to ensure that both absorb the same number of photons. The fluorescence spectra were then recorded and revealed that the emission quantum yields are of the same order, albeit significantly different (Φ_F_ = 0.17 for **14**, as compared with Φ_F_ = 0.30 [48] for the reference compound **3APH**). This sole parameter does not justify the remarkable CL variations, which have to be attributed to the lower yield of aminophthalate formation from the oxidation reaction, possibly due to the lower electron donating capability of the aromatic ring substituent. Accordingly, the perturbation of the electronic distribution of the benzenoic chromophore is reflected in the significant changes observed in the UV–vis spectra (Figure 3b).

## 3. Materials and Methods

### 3.1. General Information

All chemicals were obtained from commercial sources and were used without further purification. Solvents were dried according to published procedures [49]. The course of the reactions was followed with thin-layer chromatography (TLC), using aluminum sheets (0.2 mm) coated with silica gel 60 with fluorescence indicator (silica gel 60 F254). Purification of the products was carried out by flash column chromatography, using silica gel 60 (230−400 mesh). Nuclear magnetic resonance (NMR) spectra were obtained with a Bruker Avance 400MHz (Bruker BioSpin MRI GmbH, Ettlingen, Germany) or a Varian Mercury 200MHz spectrometer (Varian Inc., Yarnton, UK). Chemical shifts are reported in ppm. HRMS spectra were recorded in a QTOF maXis impact (Bruker) spectrometer under electron spray ionization (ESI) conditions. Fluorescence spectra were registered with a Photon Technology International (PTI) spectrofluorometer (Photon Technology International, Inc. NJ, USA), model LPS-220B equipped with a 75 W Xe lamp as a light source, also equipped with a monochromator. Monitoring of the CL was performed using the same spectrofluorometer with its own lamp switched off. The set was run in the timebased mode with the detection dialed at 425 nm. Each experiment was performed at least 10 times. Triggering the chemiluminescence: luminols were dissolved in aqueous basic solutions giving a final concentration of 7.5 μM. Then, 2 mL of each sample were introduced in a quartz cuvette and the CL was triggered by addition of 2.5 μL of H_2_O_2_ (50% *w*/*w*) and 8 μL of K_3_[Fe(CN)_6_] 75 mM while vigorously stirring. The ^1^H, ^13^C and ^31^P NMR spectra of some compounds are in the Supplementary Materials.

### 3.2. Synthetic Procedures

#### 3.2.1. Synthesis of TPP Carboxylic Acids

Triphenylphosphine (11.5 mmol) was added in a solution of bromoalkyl carboxylic acid (12 mmol) in dry acetonitrile (20 mL) and the mixture was stirred at reflux under argon for 48 h. After cooling, the solvent was evaporated and the product precipitated out upon addition of ethyl acetate (or diethyl ether). Filtration and washing with the same solvent furnished pure products [38].

(5-Carboxypentyl)triphenylphosphonium bromide (**2a**). White powder (4.83 g, 92%). ^1^H NMR (200 MHz, CDCl_3_) *δ*: 8.46 (bs, 1H, COO*H*), 7.91–7.57 (m, 15H, ArH), 3.73–3.56 (m, 2H, C*H*_2_P), 2.41–2.33 (m, 2H, C*H*_2_COOH), 1.67–1.55 (m, 6H, C*H*_2_). ^13^C NMR (50 MHz, CDCl_3_) *δ:* 176.09, 135.21 (d, *J* = 2.8 Hz, PPh_3_ para), 133.66 (d, *J* = 10.0 Hz, PPh_3_ ortho), 130.65 (d, *J* = 12.5 Hz, PPh_3_ meta), 118.11 (d, *J* = 86.0 Hz, PPh_3_ ipso), 34.25, 29.58 (d, *J* = 16.2 Hz, *C*H_2_CH_2_CH_2_P), 24.06, 22.56 (d, *J* = 51.0 Hz, *C*H_2_P), 21.99 (d, *J* = 4.1 Hz, *C*H_2_CH_2_P). ^31^P NMR (81 MHz, CDCl_3_) *δ*: 25.23. ES-MS *m*/*z* for C_24_H_26_O_2_P [M]^+^: calcd. 377.2, found 377.2.

(10-Carboxydecyl)triphenylphosphonium bromide (**2b**). White powder (5.88 g, 97%). ^1^H NMR (200 MHz, CDCl_3_) *δ*: 8.82 (bs, 1H, COO*H*), 7.80–7.60 (m, 15H, ArH), 3.66–3.44 (m, 2H, C*H*_2_P), 2.29 (t, *J* = 7.0 Hz, 2H, C*H*_2_COOH), 1.69–1.38 (m, 6H, C*H*_2_), 1.31–1.00 (m, 10H, C*H*_2_). ^13^C NMR (50 MHz, CDCl_3_) *δ*: 177.66 (C=O), 135.10 (d, *J* = 2.8 Hz, PPh_3_ para), 133.52 (d, *J* = 9.9 Hz, PPh_3_ ortho), 130.53 (d, *J* = 12.5 Hz, PPh_3_ meta), 118.09 (d, *J* = 85.9 Hz, PPh_3_ ipso), 34.40, 30.28 (d, *J* = 15.9 Hz, *C*H_2_CH_2_CH_2_P), 28.99, 28.86, 28.84, 28.82, 28.73, 24.65, 22.56 (d, *J* = 50.7 Hz, *C*H_2_P), 22.44 (d, *J* = 4.5 Hz, *C*H_2_CH_2_P). ^31^P NMR (81 MHz, CDCl_3_) *δ*: 25.08. ES-MS *m*/*z* for C_29_H_36_O_2_P [M]^+^: calcd. 447.2, found 447.2.

#### 3.2.2. Synthesis of the Nitrophthalic Anhydrides 4a,b

A mixture of 3- or 4-nitrophthalic acid (10 g, 0.047 mol) and acetic anhydride (24 mL) was stirred at reflux for 1h. After cooling, volatiles were evaporated (repeated addition of toluene and evaporation facilitated the procedure). The anhydride precipitated out of the residue upon addition of diethyl ether as sub-white powder (3-nitrophthalic anhydride **4a**: 7.90 g (90%), 4-nitrophthalic anhydride **4b**: 8.00 g (88%)). The anhydrides were used in the next step without characterization.

#### 3.2.3. Synthesis of the Nitrophthalimides 5a,b

A mixture of 3-nitrophthalic anhydride (7 g, 36.25 mmol), *sec*-butylamine (5.3 g, 72.50 mmol) and acetic acid (60 mL) was refluxed for 18 h. After cooling, volatiles were evaporated and dichloromethane (200 mL) was added. The solution was washed with aq. NaHCO_3_ (2 × 60 mL) and water (2 × 60 mL), dried (Na_2_SO_4_) and the solvent evaporated, affording the desired phthalimides.

2-(*sec*-Butyl)-4-nitroisoindoline-1,3-dione (**5a**). From 3-nitrophthalic anhydride **4a**. Beige solid, 7.92 g (88%). ^1^H NMR (200 MHz, CDCl_3_) *δ*: 8.10–8.03 (m, 2H, H-5, H-7), 7.94–7.85 (m, 1H, H-6), 4.35–4.17 (m, 1H, NCH), 2.13–1.67 (m, 2H, CH_2_), 1.45 (d, *J* = 7.0 Hz, 3H, CHC*H*_3_), 0.86 (t, *J* = 7.4 Hz, 3H, CH_3_). ^13^C NMR (50 MHz, CDCl_3_) *δ:* 165.98, 163.08, 144.91, 135.34, 133.85, 128.86, 126.82, 123.33, 49.91, 26.58, 18.10, 11.20. ES-MS *m*/*z* for C_12_H_12_N_2_O_4_ [M]^+^: calcd. 248.0, found 248.0. ES-HRMS *m*/*z* for C_12_H_12_N_2_NaO_4_ [M + Na]^+^: calcd. 248.0797, found 248.0801.

2-(*sec*-Butyl)-5-nitroisoindoline-1,3-dione (**5b**). From 3-nitrophthalic anhydride **4b**. Beige solid, 7.74 g (86%). ^1^H NMR (200 MHz, CDCl_3_) δ: 8.59 (s, 1H, H-4), 8.57 (d, *J* = 7.5 Hz, 1H, H-6), 8.00 (d, *J* = 7.5 Hz, 1H, H-7), 4.38–4.14 (m, 1H, NCH), 2.15–1.66 (m, 2H, CH_2_), 1.46 (d, *J* = 7.0 Hz, 3H, CHC*H*_3_), 0.85 (t, *J* = 7.4 Hz, 3H, CH_3_). ^13^C NMR (50 MHz, CDCl_3_) δ: 166.38, 166.07, 151.62, 136.35, 133.28, 129.14, 124.28, 118.43, 49.92, 26.69, 18.19, 11.20. ES-HRMS *m*/*z* for C_12_H_12_N_2_NaO_4_ [M + Na]^+^: calcd. 248.0797, found 248.0803.

#### 3.2.4. Synthesis of Aminophthalimides 6a,b

A stirred solution of the nitrophthalimide (1.83 g, 7.37 mmol) in methanol (30 mL) was degassed (Ar) for 30 min. 10% Pd/C (200 mg) was added, then bubbled with H_2_ for a while and the mixture was stirred under an H_2_ atmosphere (20 bar) for 18 h. The mixture was filtered through celite, washed with methanol, and the filtrate was concentrated, leaving the corresponding aminophthalimide.

4-Amino-2-(sec-butyl)isoindoline-1,3-dione (**6a**). From 4-nitrophthalimide **5a**. Yellow solid, 1.50 g (93%). ^1^H NMR (200 MHz, CDCl_3_) *δ*: 7.36 (dd, *J* = 8.3, 7.1 Hz, 1H, H-6), 7.08 (d, *J* = 7.1 Hz, 1H, H-5), 6.87 (d, *J* = 8.3 Hz, 1H, H-7), 5.41 (bs, 2H, NH_2_), 4.28–4.01 (m, 1H, NCH), 2.17–1.64 (m, 2H, CH_2_), 1.45 (d, *J* = 7.0 Hz, 3H, CHC*H*_3_), 0.87 (t, *J* = 7.4 Hz, 3H, CH_3_). ^13^C NMR (50 MHz, CDCl_3_) *δ:* 170.51*,* 168.84, 145.27, 134.82, 132.52, 120.88, 112.10, 110.92, 48.48, 26.81, 18.40, 11.23. ES-HRMS *m*/*z* for C_12_H_14_N_2_NaO_2_ [M + Na]^+^: calcd. 241.0947, found 241.0948.

5-Amino-2-(*sec*-butyl)isoindoline-1,3-dione (**6b**). From 5-nitrophthalimide **5b**. Yellow solid, 1.51 g (94%). ^1^H NMR (200 MHz, DMSO-*d*_6_) δ: 7.45 (d, *J* = 8.1 Hz, 1H, H-7), 6.89 (s, 1H, H-4), 6.78 (d, *J* = 7.9 Hz, 1H, H-6), 6.45 (s, 2H, NH_2_), 4.09–3.95 (m, 1H, CHC*H*_2_), 1.98–1.58 (m, 2H, CH_2_), 1.34 (d, *J* = 6.8 Hz, 3H, CHC*H*_3_), 0.76 (t, *J* = 7.2 Hz, 3H, CH_3_). ^13^C NMR (50 MHz, DMSO-*d*_6_): 168.55, 168.27, 155.02, 134.29, 124.81, 116.65, 116.49, 106.83, 47.77, 26.45, 18.45, 11.22. ES-HRMS *m*/*z* for C_12_H_14_N_2_NaO_2_ [M + Na]^+^: calcd. 241.0947, found 241.0948.

#### 3.2.5. General Procedure for the Acylation of Phthalimides

A solution of the carboxylic acid (11 mmol) in oxalyl chloride (10 mL) was stirred for 5 h under Ar. Then, the volatiles were evaporated to dryness under reduced pressure at room temperature. The residue was dissolved in dry dichloromethane (8 mL) and added dropwise to a cooled (0 °C) solution of the aminophthalimide (2.18 g, 10 mmol) and pyridine (1.61 mL, 20 mmol) in dichloromethane (24 mL) under Ar. The resulting mixture was stirred at r.t. for 18 h. Water (100 mL) was added, the layers were separated and the aqueous was washed with dichloromethane (2 × 40 mL). The combined organic layers were dried (Na_2_SO_4_), solvent was evaporated and the residue was subjected to column chromatography, affording the corresponding acylated phthalimide.

6-Bromo-*N*-(2-(*sec*-butyl)-1,3-dioxoisoindolin-4-yl)hexanamide (**8a**). From 6-bromohexanoic acid and 4-aminophthalimide **6a**. Chromatography with EtOAc/petroleum ether 8:1 to 4:1. Brownish oil (2.8 g, 71%). ^1^H NMR (200 MHz, CDCl_3_) *δ:* 9.60 (bs, 1H, NH), 8.73 (d, *J* = 8.4 Hz, 1H, H-5), 7.63 (t, *J* = 7.9 Hz, 1H, H-6), 7.45 (d, *J* = 7.2 Hz, 1H, H-7), 4.28–4.09 (m, 1H, CH), 3.41 (t, *J* = 6.7 Hz, 2H, BrCH*_2_*), 2.47 (t, *J* = 7.4 Hz, 2H, CH*_2_*CO), 2.12–1.65 (m, 6H), 1.60–1.42 (m, 5H), 0.86 (t, *J* = 7.4 Hz, 3H, CH_2_C*H*_3_). ^13^C NMR (50 MHz, CDCl_3_) *δ*: 172.01, 170.71, 168.05, 137.24, 135.78, 131.37, 124.61, 117.83, 115.55, 49.19, 37.71, 33.63, 32.45, 27.72, 26.90, 24.40, 18.54, 11.39. ES-HRMS *m*/*z* for C_18_H_22_BrN_2_O_3_ [M − H]^−^: calcd. 393.0819, found 393.0821.

11-Bromo-*N*-(2-(*sec*-butyl)-1,3-dioxoisoindolin-4-yl)undecanamide (**8b**). From 11-bromoundecanoic acid and 4-aminophthalimide **6a**. Chromatography with EtOAc/petroleum ether 8:1 to 4:1. Brownish oil (3.3 g, 70%). ^1^H NMR (200 MHz, CDCl_3_) *δ:* 9.58 (bs, 1H, NH), 8.74 (d, *J* = 8.4 Hz, 1H, H-5), 7.62 (t, *J* = 7.9 Hz, 1H, H-6), 7.44 (d, *J* = 7.3 Hz, 1H, H-7), 4.27–4.10 (m, 1H, CH), 3.37 (t, *J* = 6.8 Hz, 2H, BrCH*_2_*), 2.44 (t, *J* = 7.5 Hz, 2H, CH*_2_*CO), 2.08–1.65 (m, 6H), 1.45–1.19 (m, 15H), 0.86 (t, *J* = 7.4 Hz, 3H, CH_2_C*H*_3_). ^13^C NMR (50 MHz, CDCl_3_) *δ*: 172.49, 170.69, 167.93, 137.40, 135.71, 131.43, 124.64, 117.70, 115.57, 77.16, 49.20, 38.06, 34.08, 32.87, 29.41, 29.38, 29.29, 29.19, 28.78, 28.20, 26.93, 25.33, 18.50, 11.36. ES-HRMS *m*/*z* for C_23_H_32_BrN_2_O_3_ [M − H]^−^: calcd. 463.1602, found 463.1613.

6-Bromo-*N*-(2-(*sec*-butyl)-1,3-dioxoisoindolin-4-yl)hexanamide (**9a**). From 6-bromohexanoic acid and 5-aminophthalimide **6b**. Chromatography with methanol/DCM 0% to 5%. Brownish oil (2.7 g, 40%, mixture with 10 mol% of the corresponding chloride). ^1^H NMR (200 MHz, CDCl_3_) *δ:* 8.01 (dd, *J* = 8.1, 1.8 Hz, 1H, H-6), 7.96 (d, *J* = 1.5 Hz, 1H, H-4), 7.94 (bs, 1H, NH), 7.75 (d, *J* = 8.1 Hz, 1H, H-7), 4.32–4.14 (m, 1H, CH), 3.42 (t, *J* = 6.6 Hz, 2H, BrCH*_2_*), 2.47 (t, *J* = 7.3 Hz, 2H, CH*_2_*CO), 2.15–1.71 (m, 6H), 1.61–1.44 (m, 5H), 0.86 (t, *J* = 7.4 Hz, 3H, CH_2_C*H*_3_). ^13^C NMR (50 MHz, CDCl_3_) *δ*: 172.38, 168.10, 168.02, 143.71, 132.97, 126.00, 123.82, 123.75, 113.82, 48.85, 37.00, 33.33, 32.06, 27.39, 26.57, 24.29, 18.11, 11.04. ES-HRMS *m*/*z* for C_18_H_22_BrN_2_O_3_ [M − H]^−^: calcd. 393.0819, found 393.0811.

11-Bromo-*N*-(2-(*sec*-butyl)-1,3-dioxoisoindolin-5-yl)undecanamide (**9b**). From 11-bromoundecanoic acid and 5-aminophthalimide **6b**. Chromatography with methanol/DCM 0% to 5%. Brownish oil (2.7 g, 58%). ^1^H NMR (200 MHz, CDCl_3_) *δ:* 8.25 (bs, 1H, NH), 8.09 (d, *J* = 8.2 Hz, 1H, H-6), 7.97 (s, 1H, H-4), 7.74 (d, *J* = 8.1 Hz, 1H, H-7), 4.32–4.13 (m, 1H, CH), 3.38 (t, *J* = 6.7 Hz, 2H, BrCH*_2_*), 2.45 (t, *J* = 6.8 Hz, 2H, CH*_2_*CO), 2.08–1.65 (m, 6H), 1.47–1.19 (m, 15H), 0.86 (t, *J* = 7.3 Hz, 3H, CH_2_C*H*_3_). ^13^C NMR (50 MHz, CDCl_3_) *δ*: 172.16, 168.60, 168.29, 143.79, 133.45, 126.43, 124.46, 123.84, 113.86, 49.25, 37.91, 34.25, 32.86, 29.45, 29.42 (2C), 29.32, 28.80, 28.21, 26.96, 25.49, 18.54, 11.43. ES-HRMS *m*/*z* for C_23_H_32_BrN_2_O_3_ [M − H]^−^: calcd. 463.1602, found 463.1610.

#### 3.2.6. General Procedure for the Synthesis of Phosphonium Phthalimides

A solution of the bromide (1 mmol) and the phosphine (2 mmol) in dry acetonitrile (5 mL) was refluxed under Ar for 3 days. After cooling, the solvent was evaporated and the residue was subjected to column chromatography, yielding the corresponding phosphonium cation.

(6-((2-(*sec*-Butyl)-1,3-dioxoisoindolin-4-yl)amino)-6-oxohexyl)triphenylphosphonium bromide (**10a**). From bromide **8a** and triphenylphosphine. Chromatography with methanol/DCM 3 to 10%. White solid (335 mg, 51%). ^1^H NMR (200 MHz, CDCl_3_) *δ:* 9.53 (bs, 1H, NH), 8.62 (d, *J* = 8.4 Hz, H-5), 7.85–7.60 (m, 15H, ArH), 7.55 (t, *J* = 8.0 Hz, 1H, H-6), 7.39 (d, *J* = 7.2 Hz, 1H, H-7), 4.23–4.05 (m, 1H, CH), 3.80–3.65 (m, 2H, PCH*_2_*), 2.40 (t, *J* = 6.1 Hz, 2H, CH*_2_*CO), 2.07–1.60 (m, 8H), 1.39 (d, *J* = 6.9 Hz, 3H, CH_3_), 0.81 (t, *J* = 7.3 Hz, 3H, CH_2_C*H*_3_). ^13^C NMR (50 MHz, CDCl_3_) *δ*: 171.16, 169.35, 167.10, 136.11, 134.80, 134.34 (d, *J* = 2.7 Hz, PPh_3_ para), 132.74 (d, *J* = 10.0 Hz, PPh_3_ ortho),130.45, 129.76 (d, *J* = 12.5 Hz, PPh_3_ meta), 123.89, 117.21 (d, *J* = 86.0 Hz, PPh_3_ ipso), 116.90, 114.88, 48.90, 36.26, 28.82 (d, *J* = 16.0 Hz, *C*H_2_CH_2_CH_2_P), 25.94, 23.52, 21.44 (d, *J* = 50.0 Hz, CH_2_P), 21.40 (d, *J* = 4.0 Hz, *C*H_2_CH_2_P), 17.60, 10.49. ^31^P NMR (81 MHz, CDCl_3_) *δ*: 24.89. ES-HRMS *m*/*z* for C_36_H_38_N_2_O_3_P [M]^+^: calcd. 577.2615, found 577.2608.

(6-((2-(*sec*-Butyl)-1,3-dioxoisoindolin-4-yl)amino)-6-oxohexyl)tri-*p*-tolylphosphonium bromide (**10b**). From bromide **8a** and tri(*p*-tolyl)phosphine. Chromatography with methanol/DCM 5% to 20%. White solid (371 mg, 53%). ^1^H NMR (200 MHz, CDCl_3_) *δ*: 9.56 (bs, 1H, NH), 8.67 (d, *J* = 8.4 Hz, H-5), 7.70–7.57 (m, 7H, ArH), 7.50–7.43 (m, 7H, ArH, H-6, H-7), 4.26–4.08 (m, 1H, CH), 3.64–3.50 (m, 2H, PCH*_2_*), 2.46–2.34 (m, 11H, ArCH_3_, CH*_2_*CO), 2.07–1.60 (m, 8H), 1.43 (d, *J* = 6.9 Hz, 3H, CH_3_), 0.85 (t, *J* = 7.4 Hz, 3H, CH_2_C*H*_3_). ^13^C NMR (50 MHz, CDCl_3_) *δ*: 171.88, 170.24, 167.92, 146.15 (d, *J* = 3.0 Hz, PAr_3_ para), 136.94, 135.47, 133.34 (d, *J* = 10.3 Hz, PAr_3_ ortho), 131.26, 131.09 (d, *J* = 12.9 Hz, PAr_3_ meta), 124.57, 117.64, 115.59, 114.83 (d, *J* = 88.7 Hz, PAr_3_ ipso), 48.98, 37.03, 29.63 (d, *J* = 16.6 Hz, *C*H_2_CH_2_CH_2_P), 26.70, 24.35, 22.66 (d, *J* = 53.0 Hz, CH_2_P), 22.18 (d, *J* = 4.5 Hz, *C*H_2_CH_2_P), 21.79 (d, *J* = 1.2 Hz, ArCH_3_), 18.36, 11.23. ^31^P NMR (81 MHz, CDCl_3_) *δ*: 23.97. ES-HRMS *m*/*z* for C_39_H_44_N_2_O_3_P [M]^+^: calcd. 619.3084, found 619.3080.

(11-((2-(*sec*-Butyl)-1,3-dioxoisoindolin-4-yl)amino)-11-oxoundecyl)triphenylphosphonium bromide (**10c**). From bromide **8b** and triphenylphosphine. Chromatography with methanol/DCM 5% to 10%. White solid (582 mg, 80%). ^1^H NMR (200 MHz, CDCl_3_) *δ:* 9.55 (bs, 1H, NH), 8.71 (d, *J* = 8.4 Hz, H-5), 7.84–7.55 (m, 16H, ArH, H-6), 7.41 (d, *J* = 7.2 Hz, 1H, H-7), 4.24–4.07 (m, 1H, CH), 3.77–3.61 (m, 2H, PCH*_2_*), 2.39 (t, *J* = 7.5 Hz, 2H, CH*_2_*CO), 2.09–1.58 (m, 6H), 1.41 (d, *J* = 6.9 Hz, 3H, CH_3_), 1.34–1.10 (m, 12H), 0.83 (t, *J* = 7.4 Hz, 3H, CH_2_C*H*_3_). ^13^C NMR (50 MHz, CDCl_3_) *δ*: 172.55, 170.70, 168.12, 137.42, 135.71, 135.08 (d, *J* = 3.0 Hz, PPh_3_ para), 133.79 (d, *J* = 9.9 Hz, PPh_3_ ortho), 131.48, 130.58 (d, *J* = 12.6 Hz, PPh_3_ meta), 124.69, 118.25 (d, *J* = 85.9 Hz, PPh_3_ ipso), 117.72, 115.63, 49.24, 38.06, 30.49 (d, *J* = 15.5 Hz, *C*H_2_CH_2_CH_2_P), 29.37, 29.27 (2C), 29.18 (2C), 26.95, 25.32, 22.87 (d, *J* = 49.6 Hz, CH_2_P), 22.75 (d, *J* = 4.4 Hz, *C*H_2_CH_2_P), 18.52, 11.37. ^31^P NMR (81 MHz, CDCl_3_) *δ*: 25.31. ES-HRMS *m*/*z* for C_41_H_48_N_2_O_3_P [M]^+^: calcd. 647.3397, found 647.3397.

(11-((2-(*sec*-Butyl)-1,3-dioxoisoindolin-4-yl)amino)-11-oxoundecyl)tri-*p*-tolylphosphonium bromide (**10d**). From bromide **8b** and tri(*p*-tolyl)phosphine. Chromatography with methanol/DCM 5% to 10%. White solid (485 mg, 63%). ^1^H NMR (200 MHz, CDCl_3_) *δ:* 9.57 (bs, 1H, NH), 8.73 (d, *J* = 8.4 Hz, H-5), 7.68–7.42 (m, 14H, ArH, H-6, H-7), 4.26–4.09 (m, 1H, CH), 3.57–3.42 (m, 2H, PCH*_2_*), 2.44 (s, 9H, ArCH_3_), 2.39 (t, *J* = 7.9 Hz, 2H, CH*_2_*CO), 2.07–1.57 (m, 6H), 1.43 (d, *J* = 7.0 Hz, 3H, CH_3_), 1.36–1.13 (m, 12H), 0.88 (t, *J* = 7.4 Hz, 3H, CH_2_C*H*_3_). ^13^C NMR (50 MHz, CDCl_3_) *δ*: 172.55, 170.67, 168.10, 146.25 (d, *J* = 3.0 Hz, PAr_3_ para), 137.36, 135.70, 133.53 (d, *J* = 10.3 Hz, PAr_3_ ortho), 131.41, 131.21 (d, *J* = 12.9 Hz, PAr_3_ meta), 124.64, 117.71, 115.56, 115.19 (d, *J* = 88.6 Hz, PAr_3_ ipso), 49.20, 38.04, 30.55 (d, *J* = 15.6 Hz, *C*H_2_CH_2_CH_2_P), 29.76, 29.38, 29.27, 29.22, 29.17, 26.91, 25.30, 23.06 (d, *J* = 51.1 Hz, CH_2_P), 22.67 (d, *J* = 4.3 Hz, *C*H_2_CH_2_P), 21.94 (d, *J* = 1.2 Hz, ArCH_3_) 18.52, 11.37. ^31^P NMR (81 MHz, CDCl_3_) *δ*: 24.13. ES-MS m/z for C_44_H_54_N_2_O_3_P [M]^+^: calcd. 689.3867, found 689.3869.

(11-((2-(*sec*-Butyl)-1,3-dioxoisoindolin-4-yl)amino)-11-oxoundecyl)tricyclohexylphosphonium bromide (**10e**). From bromide **8b** and tricyclohexylphosphine. Chromatography with methanol/DCM 5% to 10%. White solid (723 mg, 97%). ^1^H NMR (200 MHz, CDCl_3_) *δ*: 9.51 (bs, 1H, NH), 8.65 (d, *J* = 8.3 Hz, H-5), 7.55 (t, *J* = 8.1 Hz, 1H, H-6), 7.36 (d, *J* = 7.2 Hz, 1H, H-7), 4.20–4.02 (m, 1H, CH), 2.67–2.22 (m, 7H, PCH, COCH_2_), 1.96–1.15 (m, 51H, CH), 0.78 (t, *J* = 7.3 Hz, 3H, CH_2_C*H*_3_). ^13^C NMR (50 MHz, CDCl_3_) *δ*: 172.07, 170.18, 167.63, 136.92, 135.27, 130.98, 124.19, 117.24, 115.14, 48.73, 37.59, 30.91 (d, *J* = 13.6 Hz, *C*H_2_CH_2_CH_2_P), 29.46 (d, *J* = 40.3 Hz, PCy_3_-C1), 28.91, 28.88, 28.86, 28.73, 28.61, 26.87 (d, *J* = 3.5 Hz, PCy_3_-C2), 26.46, 26.08 (d, *J* = 11.8 Hz, PCy_3_-C3), 25.08, 24.86, 22.44 (d, *J* = 5.2 Hz, *C*H_2_CH_2_P), 18.08, 15.45 (d, *J* = 43.0 Hz, CH_2_P), 10.94. ^31^P NMR (81 MHz, CDCl_3_) *δ*: 32.62. ES-HRMS *m*/*z* for C_41_H_66_N_2_O_3_P [M]^+^: calcd. 665.4806, found 665.4804.

(6-((2-(*sec*-Butyl)-1,3-dioxoisoindolin-5-yl)amino)-6-oxohexyl)tri-*p*-tolylphosphonium bromide (**11a**). From bromide **9a** and tri(*p*-tolyl)phosphine. Chromatography with methanol/DCM 3 to 10%. White solid (504 mg, 72%). ^1^H NMR (200 MHz, CDCl_3_) *δ*: 10.98 (bs, 1H, NH), 8.59 (s, 1H, H-4), 8.13 (d, *J* = 7.9 Hz, H-6), 7.67–7.43 (m, 13H, ArH, H-7), 4.29–4.10 (m, 1H, CH), 3.45–3.26 (m, 2H, PCH*_2_*), 2.65 (t, *J* = 7.2 Hz, CH*_2_*CO), 2.46 (s, 9H, ArCH_3_), 2.06–1.70 (m, 8H), 1.43 (d, *J* = 6.9 Hz, 3H, CH_3_), 0.84 (t, *J* = 7.4 Hz, 3H, CH_2_C*H*_3_). ^13^C NMR (50 MHz, CDCl_3_) *δ*: 173.10, 168.45, 168.28, 146.26 (d, *J* = 3.0 Hz, PAr_3_ para), 145.00, 133.09 (d, *J* = 10.3 Hz, PAr_3_ ortho), 132.66, 131.03 (d, *J* = 12.9 Hz, PAr_3_ meta), 125.20, 124.14, 123.14, 114.49 (d, *J* = 88.9 Hz, PAr_3_ ipso), 113.83, 48.47, 36.72, 29.70 (d, *J* = 15.9 Hz, *C*H_2_CH_2_CH_2_P), 26.61, 24.32, 22.87 (d, *J* = 52.5 Hz, CH_2_P), 21.64 (d, *J* = 1.2 Hz, ArCH_3_), 21.39 (d, *J* = 4.0 Hz, *C*H_2_CH_2_P) 18.22, 11.07. ^31^P NMR (81 MHz, CDCl_3_) *δ*: 22.24. ES-HRMS *m*/*z* for C_39_H_44_N_2_O_3_P [M]^+^: calcd. 619.3084, found 619.3094.

(6-((2-(*sec*-Butyl)-1,3-dioxoisoindolin-5-yl)amino)-6-oxohexyl)tricyclohexylphosphonium bromide (**11b**). From bromide **9a** and tricyclohexylphosphine. Chromatography with methanol/DCM 3% to 10%. White solid (595 mg, 88%, contaminated with 25 mol% tricyclohexylphosphinoxide). ^1^H NMR (400 MHz, CDCl_3_) *δ*: 10.87 (bs, 1H, NH), 8.42 (d, *J* = 1.8 Hz, 1H, H-4), 8.04 (dd, *J* = 8.2, 1.9 Hz, H-6), 7.45 (d, *J* = 8.1 Hz, 1H, H-7), 4.06–3.97 (m, 1H, NCH), 2.57 (t, *J* = 7.3 Hz, CH*_2_*CO), 2.48–2.39 (m, 3H, PCH), 2.18–2.11 (m, 2H, PCH_2_), 1.97–1.05 (m, 41H, CH, O=CCy_3_*), 0.67 (t, *J* = 7.4 Hz, 3H, CH_2_C*H*_3_). ^13^C NMR (100 MHz, CDCl_3_) *δ*: 172.85, 168.33, 168.20, 144.95, 132.66, 125.25, 123.98, 123.09, 113.64, 48.45, 36.11, 35.02* (d, *J* = 60.8 Hz, O=PCy_3_-C1), 30.00 (d, *J* = 14.1 Hz, *C*H_2_CH_2_CH_2_P), 29.64 (d, *J* = 40.4 Hz, PCy_3_-C1), 26.90 (d, *J* = 3.8 Hz, PCy_3_-C2), 26.59* (d, *J* = 11.6 Hz, O=PCy_3_-C3), 26.55, 26.12 (d, *J* = 11.9 Hz, PCy_3_-C3), 26.02* (d, *J* = 3.0 Hz, O=PCy_3_-C2), 25.82* (d, *J* = 1.3 Hz, O=PCy_3_-C4), 25.07 (d, *J* = 1.2 Hz, PCy_3_-C4), 24.20 (d, *J* = 1.0 Hz, *C*H_2_CH_2_CH_2_CH_2_P), 21.43 (d, *J* = 4.7 Hz, *C*H_2_CH_2_P), 18.13, 15.48 (d, *J* = 42.8 Hz, CH_2_P), 10.96. ^31^P NMR (81 MHz, CDCl_3_) *δ*: 33.64. ES-HRMS *m*/*z* for C_36_H_56_N_2_O_3_P [M]^+^: calcd. 595.4023, found 595.4032. *signals attributed to tricyclohexylphosphinoxide.

(11-((2-(*sec*-Butyl)-1,3-dioxoisoindolin-5-yl)amino)-11-oxoundecyl)tri-*p*-tolylphosphonium bromide (**11c**). From bromide **9b** and tri(*p*-tolyl)phosphine. Chromatography with methanol/DCM 3 to 10%. White solid (523 mg, 68%). ^1^H NMR (400 MHz, CDCl_3_) *δ*: 10.74 (bs, 1H, NH), 8.52 (d, *J* = 1.8 Hz, 1H, H-4), 8.14 (dd, *J* = 8.2, 1.8 Hz, H-6), 7.51 (dd, *J* = 12.4, 8.1 Hz, PTol-H_ortho_), 7.46 (d, *J* = 8.1 Hz, 1H, H-7), 7.41 (dd, *J* = 8.3, 3.2 Hz, PTol-H_meta_), 4.13–4.04 (m, 1H, NCH), 3.29–3.22 (m, 1H, PCH), 2.58 (t, *J* = 7.5 Hz, CH_2_CO), 2.39 (s, PTol-CH_3_), 1.97–1.85 (m, 1H, C*H*_2_CH_3_), 1.70–1.43 (m, 7H), 1.32 (t, *J* = 7.0, CHC*H*_3_), 1.27–1.10 (m, 10H), 0.74 (t, *J* = 7.4 Hz, 3H, CH_2_C*H*_3_). ^13^C NMR (100 MHz, CDCl_3_) *δ*: 173.82, 168.69, 168.51, 146.48 (d, *J* = 2.9 Hz, PAr_3_ para), 145.42, 133.26 (d, *J* = 10.4 Hz, PAr_3_ ortho), 132.85, 131.20 (d, *J* = 12.8 Hz, PAr_3_ meta), 125.24, 124.28, 123.29, 114.84 (d, *J* = 88.7 Hz, PAr_3_ ipso), 114.14, 48.64, 37.10, 30.28 (d, *J* = 15.5 Hz, *C*H_2_CH_2_CH_2_P), 28.75, 28.71, 28.70, 28.65, 28.39, 26.82, 25.29, 23.07 (d, *J* = 51.9 Hz, CH_2_P), 22.39 (d, *J* = 4.4 Hz, *C*H_2_CH_2_P), 21.79 (d, *J* = 1.5 Hz, ArCH_3_), 18.37, 11.21. ^31^P NMR (162 MHz, CDCl_3_) *δ*: 22.81. ES-HRMS *m*/*z* for C_44_H_54_N_2_O_3_P [M]^+^: calcd. 689.3867, found 689.3886.

#### 3.2.7. General Procedure for the Synthesis of Phosphonium Phthalhydrazides

A solution of the phosphonium phthalimide (0.4 mmol) and hydrazine hydrate (6 mmol) in ethanol (15 mL) was refluxed for 3 h. After cooling, the volatiles were evaporated and the residue was subjected to column chromatography, yielding the corresponding phthalhydrazide.

(6-((1,4-Dioxo-1,2,3,4-tetrahydrophthalazin-5-yl)amino)-6-oxohexyl)triphenylphosphonium bromide (**1a**). From phthalimide **10a**. Chromatography with methanol/DCM 5% to 20%. White solid (139 mg, 53%). ^1^H NMR (200 MHz, CDCl_3_) *δ*: 12.53 (bs, 1H, NH), 12.06 (bs, 2H, NH),8.78 (d, *J* = 8.1 Hz, 1H, H-6), 7.80–7.45 (m, 17H, Ar-H, H-7, H-8), 3.71–3.50 (m, 2H, CH_2_P), 2.39–2.22 (m, 2H, C*H_2_*CO), 1.78–1.54 (m, 6H, C*H*_2_). ^13^C NMR (50 MHz, CDCl_3_) *δ*: 172.02, 159.90, 154.47, 140.90, 135.13 (d, *J* = 1.8 Hz, PAr_3_ para), 134.21, 133.52 (d, *J* = 9.9 Hz, PAr_3_ ortho), 130.54 (d, *J* = 12.5 Hz, PAr_3_ meta), 127.91, 122.22, 119.59, 118.03 (d, *J* = 85.9 Hz, ipso), 114.80, 37.95, 29.71 (d, *J* = 17.4 Hz, *C*H_2_CH_2_CH_2_P), 24.40, 22.25 (d, *J* = 49.6 Hz, CH_2_P), 22.23 (d, *J* = 3.2 Hz, *C*H_2_CH_2_P). ^31^P NMR (81 MHz, CDCl_3_) *δ*: 25.33. ES-HRMS *m*/*z* for C_32_H_31_N_3_O_3_P [M]^+^: calcd. 536.2098, found 536.2097.

(6-((1,4-Dioxo-1,2,3,4-tetrahydrophthalazin-5-yl)amino)-6-oxohexyl)tri-p-tolylphosphonium bromide (**1b**). From phthalimide **10b**. Chromatography with methanol/DCM 5% to 20%. White solid (84 mg, 32%). ^1^H NMR (200 MHz, CDCl_3_) *δ*: 12.53 (bs, 1H, NH), 12.66 (bs, 2H, NH), 8.80 (d, *J* = 8.0 Hz, 1H, H-6), 7.70 (d, *J* = 7.0 Hz, 1H, H-8), 7.57–7.30 (m, 13H, Ar-H, H-7), 3.46–3.25 (m, 2H, CH_2_P), 2.41–2.18 (m, 11H, ArCH_3_, CH_2_CO), 1.78–1.52 (m, 6H, C*H*_2_). ^13^C NMR (50 MHz, CDCl_3_) *δ*: 172.34, 160.21, 155.19, 146.48 (d, *J* = 1.6 Hz, PAr_3_ para), 140.93, 134.08, 133.44 (d, *J* = 10.4 Hz, PAr_3_ ortho), 131.30 (d, *J* = 12.9 Hz, PAr_3_ meta),128.51, 122.31, 119.94, 114.98 (d, *J* = 88.8 Hz, PAr_3_ ipso), 115.25, 38.29, 29.93 (d, *J* = 16.0 Hz, *C*H_2_CH_2_CH_2_P), 24.58, 22.74 (d, *J* = 49.2 Hz, CH_2_P), 22.30 (d, *J* = 4.8 Hz, *C*H_2_CH_2_P), 21.97. ^31^P NMR (81 MHz, CDCl_3_) *δ*: 25.23. ES-HRMS *m*/*z* for C_35_H_37_N_3_O_3_P [M]^+^: calcd. 578.2657, found 578.2619.

(11-((1,4-Dioxo-1,2,3,4-tetrahydrophthalazin-5-yl)amino)-11-oxoundecyl)triphenylphosphonium bromide (**1c**). From phthalimide **10c**. Chromatography with methanol/DCM 5% to 20%. White solid (135 mg, 49%). ^1^H NMR (200 MHz, DMSO-*d*_6_) *δ*: 15.58 (bs, 1H, NH), 8.74 (d, *J* = 7.7 Hz, 1H, H-6), 7.90–7.70 (m, 15H, ArH), 7.62 (d, *J* = 7.7 Hz, 1H, H-8), 7.49 (t, *J* = 7.7 Hz, 1H, H-7), 3.60–3.45 (m, 2H, CH_2_P), 2.28 (t, *J* = 7.1 Hz, 2H, CH*_2_*CO), 1.65–1.10 (m, 16H, CH_2_). ^13^C NMR (50 MHz, DMSO-*d*_6_) *δ*: 171.09, 160.37, 156.81, 140.29, 134.84 (d, *J* = 2.6 Hz, PAr_3_ para), 133.58 (d, *J* = 10.1 Hz, PAr_3_ ortho), 130.34, 130.22 (d, *J* = 12.4 Hz, PAr_3_ meta), 129.99, 119.50, 118.62 (d, *J* = 85.6 Hz, PAr_3_ ipso), 118.59, 117.36, 38.10, 30.72, 29.67 (d, *J* = 16.5 Hz, *C*H_2_CH_2_CH_2_P), 28.59 (2C), 28.51, 27.95, 25.03, 21.67 (d, *J* = 4.2 Hz, *C*H_2_CH_2_P), 20.09 (d, *J* = 49.4 Hz, CH_2_P). ^31^P NMR (162 MHz, CDCl_3_) *δ*: 23.39. ES-HRMS *m*/*z* for C_37_H_41_N_3_O_3_P [M]^+^: calcd. 606.2880, found 606.2888.

(11-((1,4-Dioxo-1,2,3,4-tetrahydrophthalazin-5-yl)amino)-11-oxoundecyl)tri-*p*-tolylphosphonium bromide (**1d**). From phthalimide **10d**. Chromatography with methanol/DCM 5% to 20%. White solid (108 mg, 37%). ^1^H NMR (200 MHz, CDCl_3_) *δ*: 12.74 (bs, 1H, NH), 8.95 (d, *J* = 8.2 Hz, 1H, H-6), 7.77 (d, *J* = 7.7 Hz, 1H, H-8), 7.62 (t, *J* = 8.1 Hz, 1H, H-7), 7.55–7.36 (m, 15H, ArH), 3.36–3.22 (m, 2H, CH_2_P), 2.42–2.27 (m, 11H, ArCH_3_, CH_2_CO), 1.72–1.04 (m, 16H, CH_2_). ^13^C NMR (50 MHz, CDCl_3_) *δ*: 172.80, 159.66, 154.97, 146.34 (d, *J* = 3.0 Hz, PAr_3_ para), 141.21, 134.06, 133.24 (d, *J* = 10.3 Hz, PAr_3_ ortho), 131.15 (d, *J* = 12.9 Hz, PAr_3_ meta), 128.28, 122.41, 119.66, 115.12, 114.86 (d, *J* = 88.7 Hz, PAr_3_ ipso), 38.62, 30.38 (d, *J* = 15.7 Hz, *C*H_2_CH_2_CH_2_P), 28.91 (2C), 28.87, 28.83, 28.77, 25.31, 22.82 (d, *J* = 51.6 Hz, CH_2_P), 22.49 (d, *J* = 4.2 Hz, *C*H_2_CH_2_P), 21.81 (d, *J* = 1.2 Hz, ArCH_3_). ^31^P NMR (81 MHz, CDCl_3_) *δ*: 23.82. ES-HRMS *m*/*z* for C_40_H_47_N_3_O_3_P [M]^+^: calcd. 648.3350, found 648.3437.

Tricyclohexyl(11-((1,4-dioxo-1,2,3,4-tetrahydrophthalazin-5-yl)amino)-11-oxoundecyl)phosphonium bromide (**1e**). From phthalimide **10e**. Chromatography with methanol/DCM 5% to 20%. White solid (56 mg, 20%). ^1^H NMR (400 MHz, CDCl_3_) *δ*: 12.67 (bs, 1H, NH), 8.98 (d, *J* = 8.3 Hz, 1H, H-6), 7.81 (d, *J* = 7.8 Hz, 1H, H-8), 7.69 (t, *J* = 8.1 Hz, 1H, H-7), 2.53 (q, *J* = 11.5 Hz, 3H, PCyCH), 2.40 (t, *J* = 7.5 Hz, 2H, COCH_2_), 2.38–2.30 (m, 2H, PCH_2_), 2.01–1.19 (m, 46H, CH_2_). ^13^C NMR (100 MHz, CDCl_3_) *δ*: 172.81, 159.82, 154.77, 141.40, 134.35, 128.09, 122.63, 119.67, 115.09, 38.73, 31.18 (d, *J* = 13.6 Hz, *C*H_2_CH_2_CH_2_P), 30.02 (d, *J* = 40.3 Hz, PCy_3_-C1), 29.15, 28.98, 28.94, 28.86, 28.82, 27.25 (d, *J* = 3.9 Hz, PCy_3_-C2), 26.53 (d, *J* = 11.7 Hz, PCy_3_-C3), 25.47, 25.35, 22.79 (d, *J* = 5.2 Hz, *C*H_2_CH_2_P), 15.82 (d, *J* = 42.5 Hz, CH_2_P). ^31^P NMR (81 MHz, CDCl_3_) *δ*: 32.71. ES-HRMS *m*/*z* for C_37_H_59_N_3_O_3_P [M]^+^: calcd. 624.4289, found 624.4295.

(6-((1,4-dioxo-1,2,3,4-tetrahydrophthalazin-6-yl)amino)-6-oxohexyl)tri-*p*-tolylphosphonium bromide (**12a**). From phthalimide **11a**. Chromatography with methanol/DCM 5% to 20%. White solid (190 mg, 72%). ^1^H NMR (200 MHz, DMSO-*d*_6_) *δ*: 11.46 (bs, 2H, NHNH), 10.63 (bs, 1H, NHCO), 8.42 (s, 1H, H-5), 8.03–7.94 (m, 2H, H-7, H-8), 7.68–7.55 (m, 12H, ArH), 3.56–3.45 (m, 2H, CH_2_P), 2.46–2.30 (m, 11H, ArCH_3_, CH*_2_*CO), 1.60–1.45 (m, 6H, CH_2_). ^13^C NMR (50 MHz, DMSO-*d*_6_) *δ*: 172.13, 155.00, 154.50, 145.53 (d, *J* = 2.8 Hz, PAr_3_ para), 143.10, 133.46 (d, *J* = 10.4 Hz, PAr_3_ ortho), 130.80 (d, *J* = 12.8 Hz, PAr_3_ meta),128.18, 126.24, 123.29, 122.33, 115.51 (d, *J* = 88.2 Hz, PAr_3_ ipso), 113.28, 36.08, 29.51 (d, *J* = 17.5 Hz, *C*H_2_CH_2_CH_2_P), 24.30, 21.67 (d, *J* = 5.0 Hz, *C*H_2_CH_2_P), 21.28 (d, *J* = 1.4 Hz, ArCH_3_), 20.52 (d, *J* = 52.0 Hz, CH_2_P). ^31^P NMR (81 MHz, DMSO-*d*_6_) *δ*: 24.04. ES-HRMS *m*/*z* for C_35_H_37_N_3_O_3_P [M]^+^: calcd. 578.2567, found 578.2627.

Tricyclohexyl(6-((1,4-dioxo-1,2,3,4-tetrahydrophthalazin-6-yl)amino)-6-oxohexyl)phosphonium bromide (**12b**). From phthalimide **11b**. Chromatography with methanol/DCM 5% to 20%. White solid (157 mg, 62%). ^1^H NMR (200 MHz, DMSO-*d*_6_) *δ*: 11.25 (bs, 1H, NHCO), 8.56 (s, 1H, H-5), 8.11 (d, *J* = 8.7 Hz, 1H, H-7), 7.97 (d, *J* = 8.6 Hz, 1H, H-8), 2.59–2.43 (m, 5H, PCH), 2.32–2.15 (m, 2H, COCH_2_), 2.03–1.26 (m, 36H, CH_2_). ^13^C NMR (50 MHz, DMSO-*d*_6_) *δ*: 172.31, 155.00, 154.39, 143.20, 128.18, 126.15, 123.34, 122.35, 113.32, 36.03, 30.11 (d, *J* = 13.3 Hz, *C*H_2_CH_2_CH_2_P), 28.54 (d, *J* = 41.2 Hz, CH_2_P-cyclo), 26.11 (d, *J* = 4.2 Hz, *C*H_2_CH_2_P-cyclo), 25.94 (d, *J* = 12.8 Hz, *C*H_2_CH_2_CH_2_P-cyclo), 25.02, 24.26, 21.44 (d, *J* = 2.8 Hz, *C*H_2_CH_2_P), 14.28 (d, *J* = 44.0 Hz, CH_2_P). ^31^P NMR (81 MHz, DMSO-*d*_6_) *δ*: 32.47. ES-HRMS *m*/*z* for C_32_H_49_N_3_O_3_P [M]^+^: calcd. 554.3506, found 554.3556.

(11-((1,4-Dioxo-1,2,3,4-tetrahydrophthalazin-6-yl)amino)-11-oxoundecyl)tri-p-tolylphosphonium bromide (**12c**). From phthalimide **11c**. Chromatography with methanol/DCM 5% to 20%. White solid (108 mg, 37%). ^1^H NMR (200 MHz, DMSO-*d*_6_) *δ*: 11.44 (bs, 2H, NHNH), 10.47 (bs, 1H, NHCO), 8.40 (s, 1H, H-5), 8.03–7.94 (m, 2H, H-7, H-8), 7.68–7.53 (m, 12H, ArH), 3.52–3.43 (m, 2H, CH_2_P), 2.44–2.33 (m, 11H, ArCH_3_, CH*_2_*CO), 1.65–1.16 (m, 16H, CH_2_). ^13^C NMR (50 MHz, DMSO-*d*_6_) *δ*: 172.32, 154.93, 154.38, 145.55 (d, *J* = 3.0 Hz, PAr_3_ para), 143.17, 133.44 (d, *J* = 10.4 Hz, PAr_3_ ortho), 130.80 (d, *J* = 12.8 Hz, PAr_3_ meta), 128.15, 126.24, 123.31, 122.23, 115.54 (d, *J* = 88.2 Hz, PAr_3_ ipso), 113.26, 36.45, 29.79 (d, *J* = 16.6 Hz, *C*H_2_CH_2_CH_2_P), 28.75, 28.68, 28.63, 28.13, 25.02, 21.76 (d, *J* = 3.5 Hz, *C*H_2_CH_2_P), 21.28 (d, *J* = 1.5 Hz, ArCH_3_), 20.46 (d, *J* = 50.4 Hz, CH_2_P). ^31^P NMR (81 MHz, DMSO-*d*_6_) *δ*: 24.13. ES-HRMS *m*/*z* for C_40_H_47_N_3_O_3_P [M]^+^: calcd. 648.3350, found 648.3401.

#### 3.2.8. Synthesis of 3-Heptanamidophthalic Acid (14)

*Synthesis of 4-aminoisobenzofuran-1,3-dione***15** [50]. A stirred solution of 3-nitrophthalic anhydride **4a** (4.5 g, 23 mmol) in THF (40 mL) was degassed (Ar) for 30 min. 10% Pd/C (200 mg) was added, then bubbled with H_2_ for a while and the mixture was stirred under H_2_ atmosphere (20 bar) for 18 h. The mixture was filtered through celite, washed with ethyl acetate and the filtrate was concentrated. The solid residue was washed with dichloromethane (extracting ≈ 1 g of a mixture containing the product), silica gel was added in the combined washings, the solvent was evaporated and the residue was dry-loaded onto column chromatography (dichloromethane), affording 3-aminophthalic anhydride **15** as yellow solid (488 mg, 13%). ^1^H NMR (200 MHz, DMSO-*d*_6_) *δ*: 7.57 (t, *J* = 7.8 Hz, 1H, H-6), 7.11 (d, *J* = 7.0 Hz, 1H, H-5), 7.09 (d, *J* = 8.5 Hz, 1H, H-4), 6.83 (s, 2H, NH_2_). ^13^C NMR (50 MHz, DMSO-*d*_6_) *δ*: 164.00, 163.92, 148.33, 137.21, 131.47, 122.25, 112.68, 108.01. ES-MS *m*/*z* for C_8_H_5_NO_3_ [M]^+^: calcd. 163.0, found 163.0. A solution of heptanoic acid (263 mg, 2.024 mmol) in oxalyl chloride (1 mL) was stirred for 5 h under Ar. Then, the volatiles were evaporated to dryness under reduced pressure at room temperature. The residue was dissolved in dry dichloromethane (1 mL) and added dropwise to a cooled (0 °C) solution of the anhydride **15** (300 mg, 1.84 mmol) and pyridine (474 μL, 5.52 mmol) in dichloromethane (5 mL) under Ar. The resulting mixture was stirred at r.t. for 48 h. Water (40 mL) was added and the aqueous phase was washed with dichloromethane (2 × 20 mL) and ethyl acetate (3 × 20 mL). The combined ethyl acetate washings were dried (Na_2_SO_4_), solvent was evaporated and the residue was subjected to column chromatography (0% to 20% methanol/DCM) yielding phthalic acid **14** as off-white solid (17 mg, 3%). ^1^H NMR (200 MHz, DMSO-*d*_6_) *δ*: 11.92 (bs, 1H, NH), 8.40 (d, *J* = 8.2, Hz, 1H, H-4), 7.38–7.27 (m, 2H, H-5, H-6), 2.28 (t, *J* = 7.3 Hz, 2H, COCH_2_), 1.64–1.52 (m, 2H, COCH_2_C*H*_2_), 1.33–1.24 (m, 6H), 0.86 (t, *J* = 6.3 Hz, 3H, hexyl-CH_3_). ^13^C NMR (50 MHz, DMSO-*d*_6_) *δ*: 173.29, 173.20, 172.82, 155.51, 135.85, 128.47, 127.95, 123.88, 123.34, 37.27, 31.31, 28.58, 25.34, 22.18, 12.98. ES-HRMS *m*/*z* for C_15_H_18_NO_5_ [M − H]^−^: calcd. 292.1185, found 292.1188.

## 4. Conclusions

A series of phosphonium-functionalized amino-acylated luminol and isoluminol derivatives was synthesized. Direct acylation of (iso)luminol resulted in inseparable mixtures, so their preparation was accomplished through the acylation of the corresponding, easily-accessible phthalimides, followed by hydrazinolysis. In this way, the targeted derivatives were isolated in a scalable and repeatable manner. All compounds were fully characterized using NMR and HRMS spectroscopies. The H_2_O_2_-triggered chemiluminescence of the synthesized compounds was investigated under alkaline conditions and compared to that of the parent compound. In general, all amino-acylated luminol derivatives exhibit a CL quantum yield markedly lower to that of luminol. The remarkable decrease in the CL quantum yield is attributed to both the weaker fluorescence of the corresponding phthalates and, more importantly, to the poorer electron-donating nature of the aromatic ring substituent, which results in a lower yield of the CL-triggering oxidation reaction. Thus, while the task of attaching a phosphonium cation on luminol is feasible, the amino-acylation approach, towards (iso)luminol functionalization, affords inefficient chemiluminescent derivatives. As a result, different functionalization routes have to be considered.

## Figures and Tables

**Table 1:** CL quantum yields of phthalhydrazides **1**, **12**, and luminol

Compound	Φ_CL_
**Luminol**	0.012 ^a^
**1a**	0.001
**1b**	0.001
**1c**	0.001
**1d**	0.001
**1e**	0.001
**12a**	< 0.001
**12b**	< 0.001
**12c**	< 0.001

^a^ See [46].

## Supplementary Materials

NMR spectra for the prepared compounds are available online at https://www.mdpi.com/1420-3049/24/21/3957/s1.
