# Supplementary material for: Synthesis and Chemiluminescent Properties of Amino-Acylated luminol Derivatives Bearing Phosphonium Cations
Source: Molecules. 2019 Oct 31;24(21):3957. doi: 10.3390/molecules24213957 (PMC6865176; doi:10.3390/molecules24213957)
Supplement: Supplementary file 1 [file molecules-24-03957-s001.pdf]

Electronic Supporting Information

for

Synthesis and chemiluminescent properties of amino-acylated luminol derivatives bearing phosphonium cations

Anna Pantelia [1], Ira Daskalaki [1], M. Consuelo Cuquerella [2], Georgios Rotas [1], Miguel A. Miranda [2,*] and Georgios C. Vougioukalakis [1,*]

[1] Laboratory of Organic Chemistry, Department of Chemistry, National and Kapodistrian University of Athens, Panepistimiopolis, 15771 Athens, Greece; annapantelia@gmail.com (A.P); iradask@hotmail.com (I.D); rotasgiorgos@hotmail.com (G.R)

[2] Instituto de Tecnología Química (UPV-CSIC), Universitat Politècnica de València, 46022 València, Spain; mcuquere@itq.upv.es (M.C.C)

* Correspondence: vougiouk@chem.uoa.gr. Tel.: +30 210 7274230. Fax: +30 210 7274761 (G.C.V); mmiranda@qim.upv.es. Tel.: +34 963 877 807. Fax: +34 963 879 444 (M.A.M)

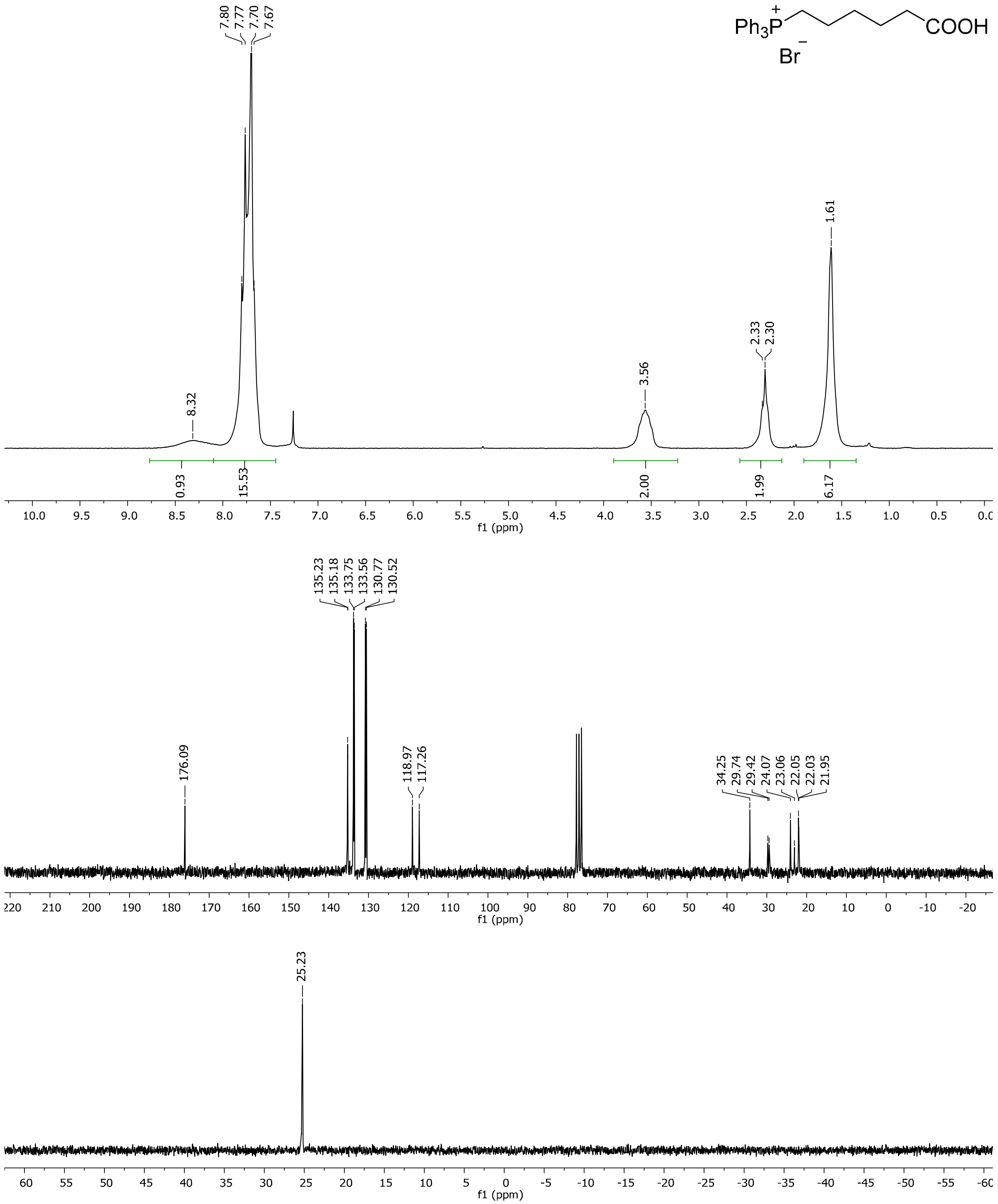

Figure S1. ^{1}H (200 MHz, top), ^{13}C (50 MHz, middle) and ^{31}P (81 MHz, bottom) NMR ($CDCl_3$) spectra of **2a**.

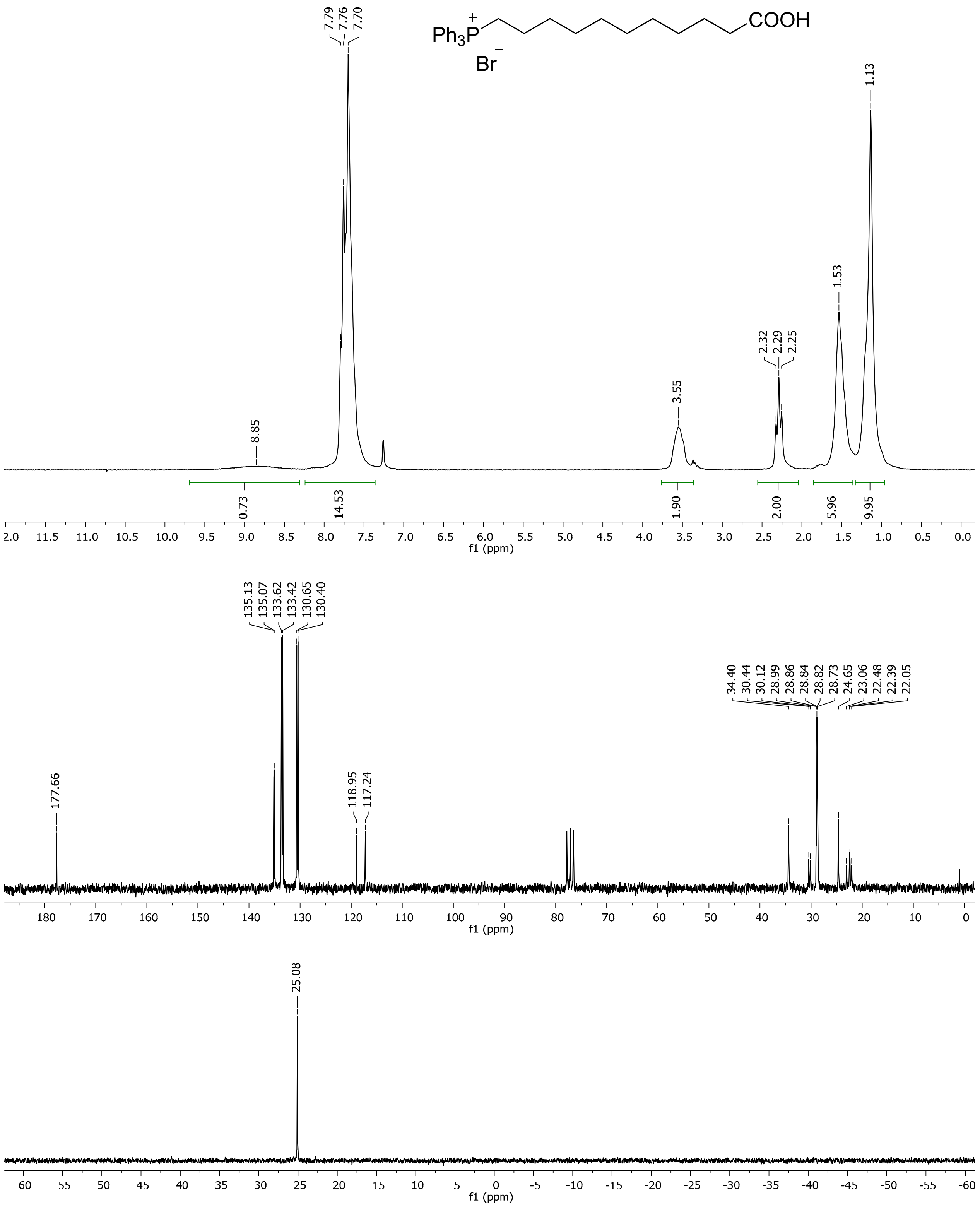

Figure S2. ^{1}H (200 MHz, top), ^{13}C (50 MHz, middle) and ^{31}P (81 MHz, bottom) NMR ($CDCl_3$) spectra of **2b**.

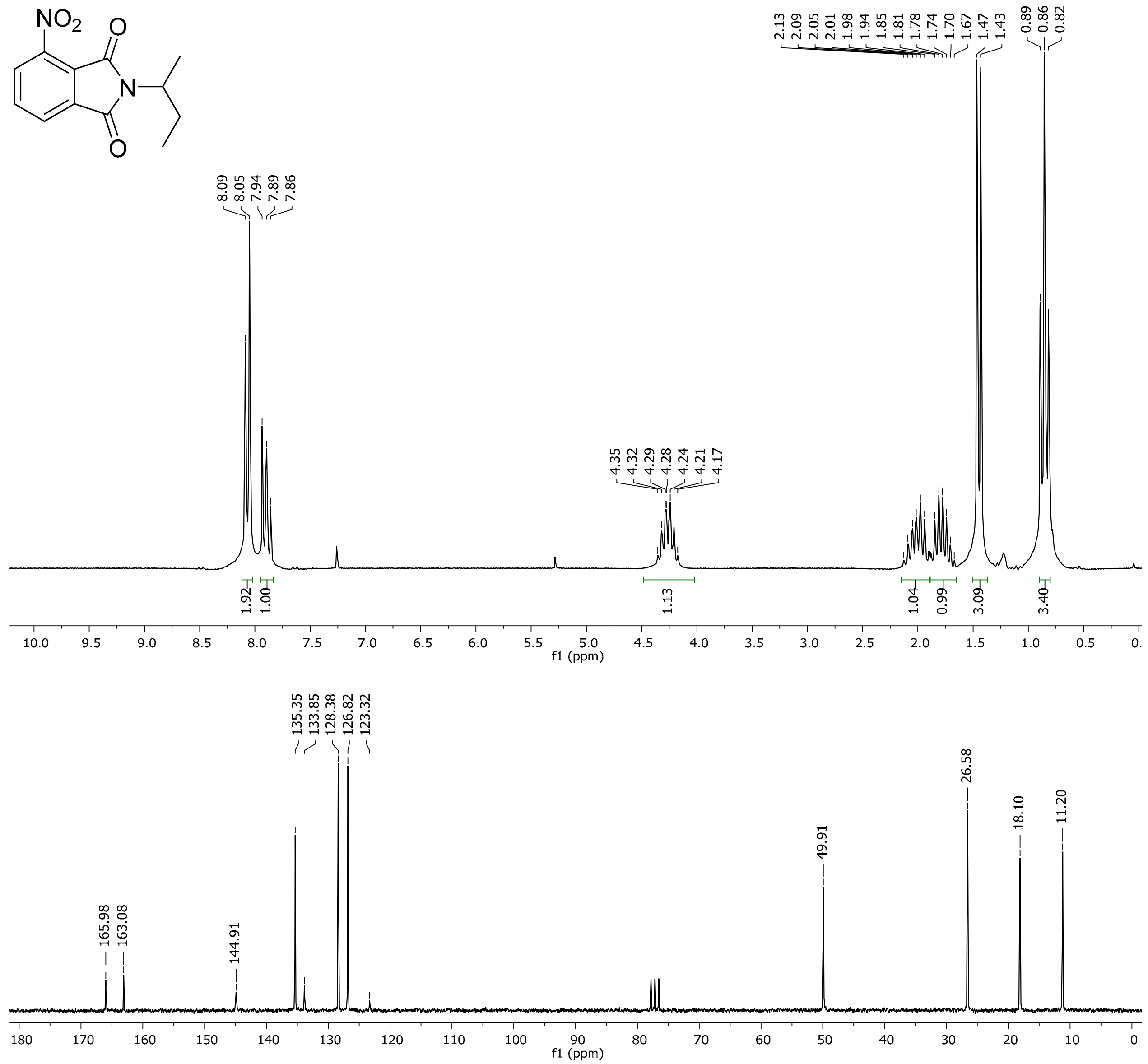

Figure S3. ^{1}H (200 MHz, top) and ^{13}C (50 MHz, bottom) NMR ($CDCl_3$) spectra of **5a**.

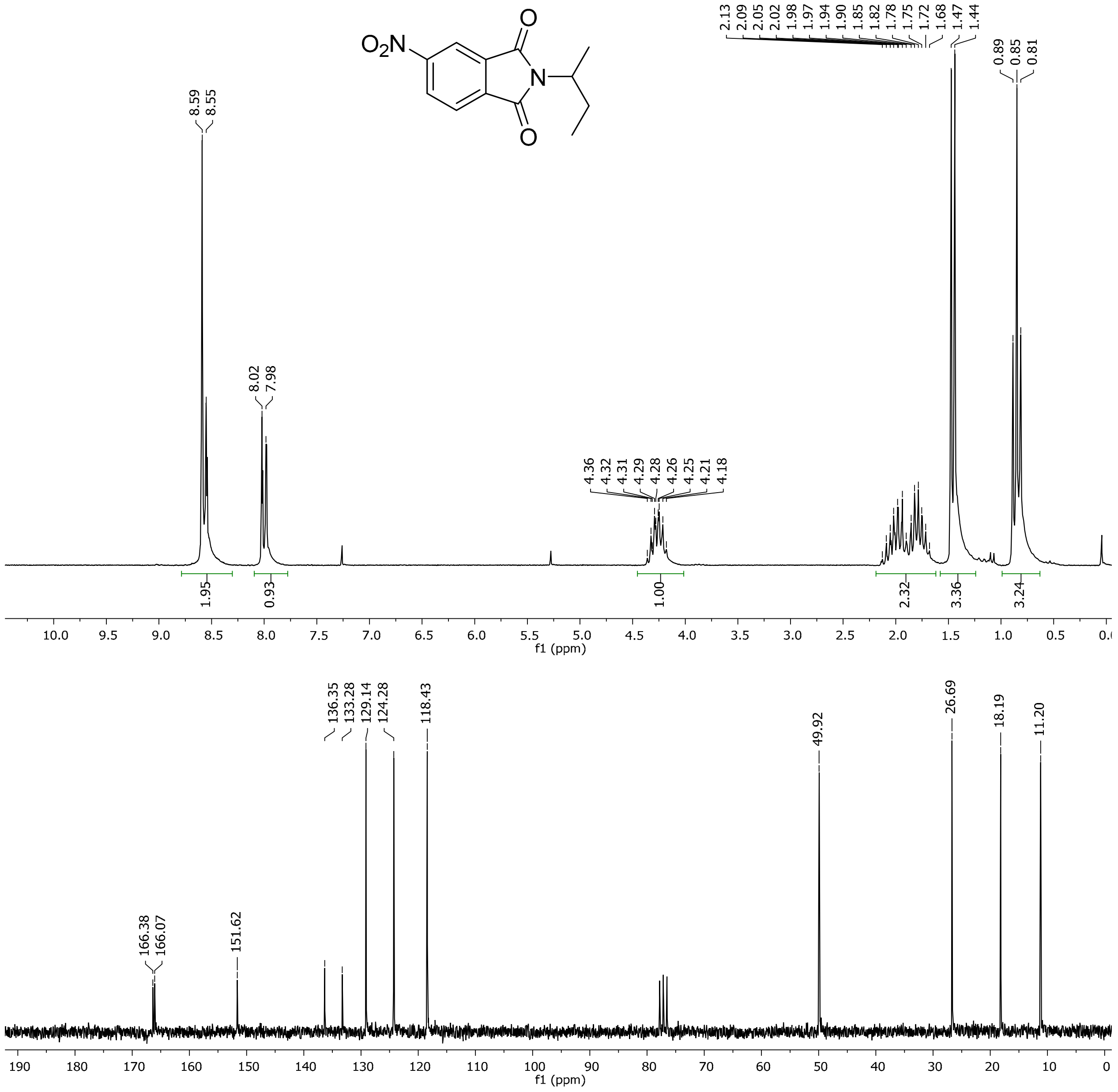

Figure S4. ^{1}H (200 MHz, top) and ^{13}C (50 MHz, bottom) NMR ($CDCl_3$) spectra of **5b**.

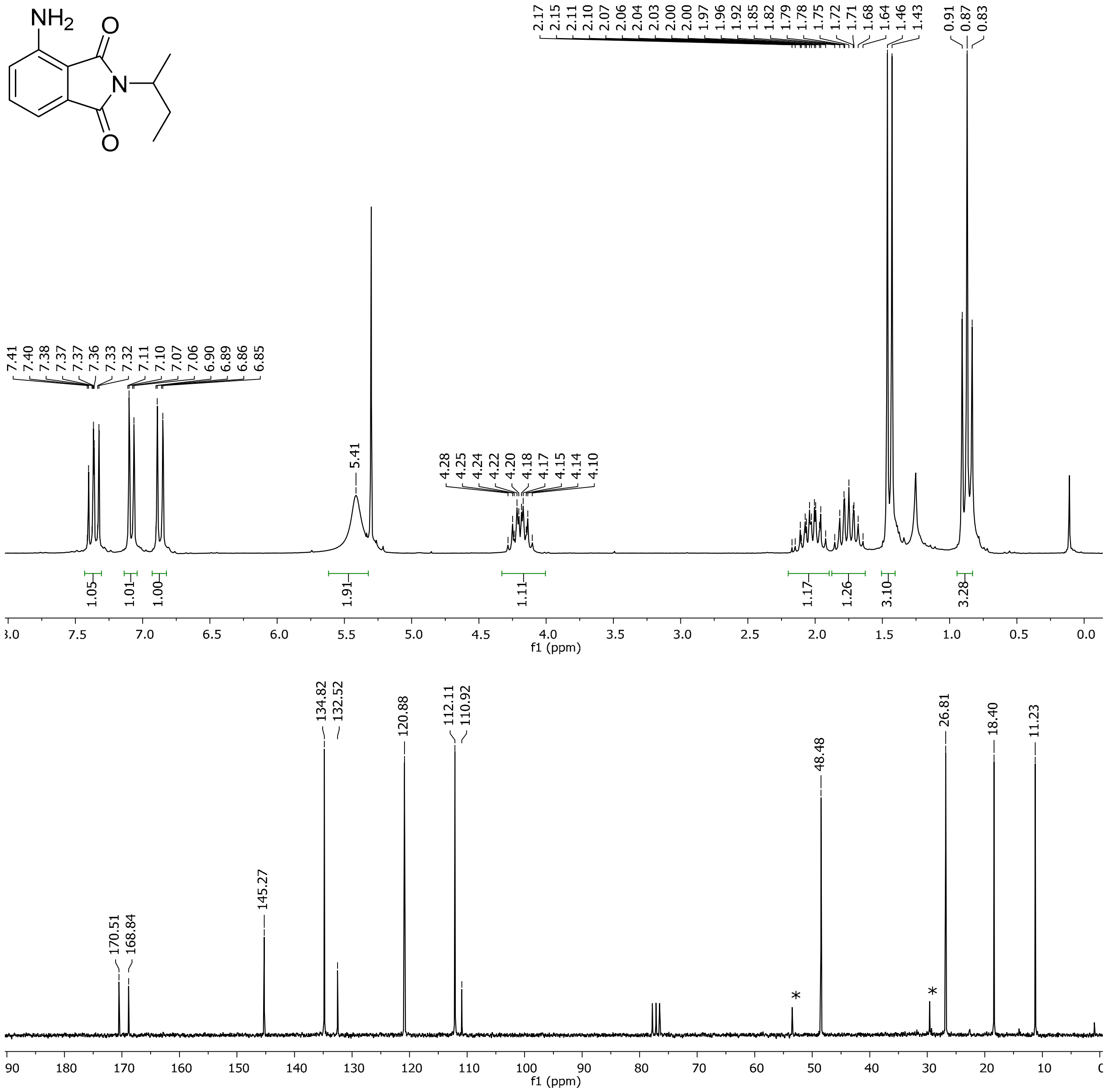

Figure S5. ^{1}H (200 MHz, top) and ^{13}C (50 MHz, bottom) NMR ($CDCl_3$) spectra of **6a**.

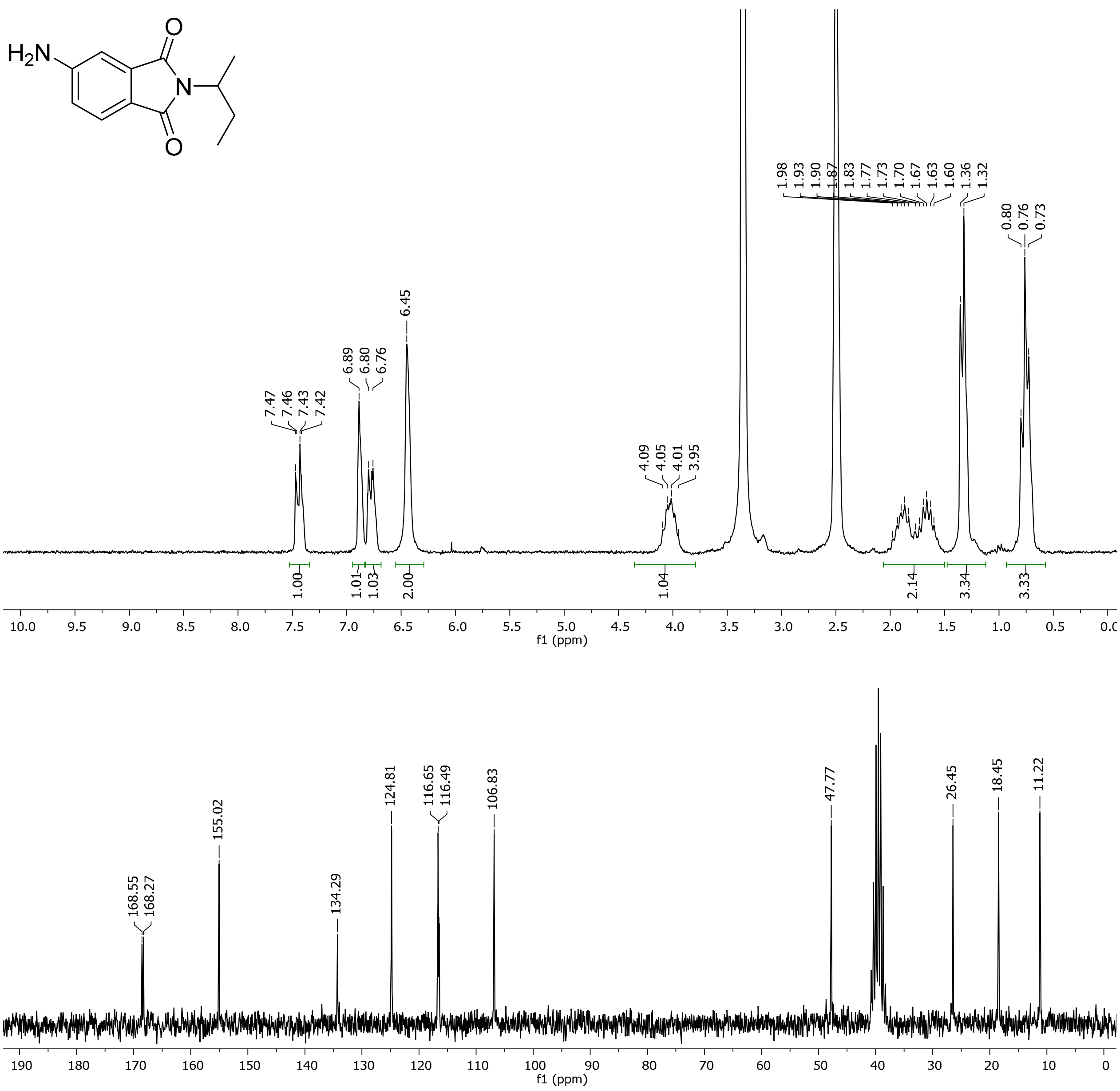

Figure S6. ^{1}H (200 MHz, top) and ^{13}C (50 MHz, bottom) NMR (DMSO-d_6) spectra of **6b**.

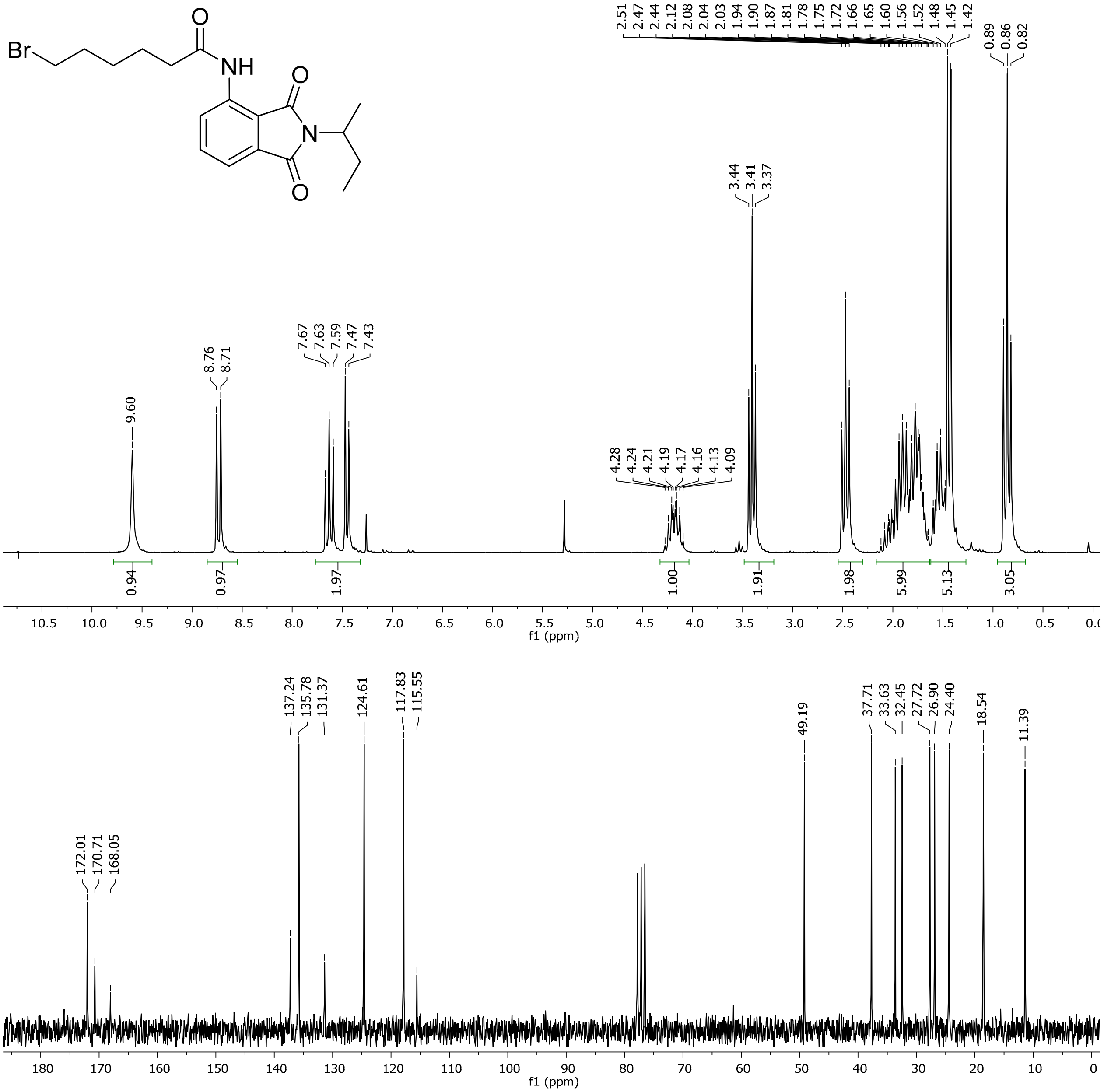

Figure S7. ^{1}H (200 MHz, top) and ^{13}C (50 MHz, bottom) NMR ($CDCl_3$) spectra of **8a**.

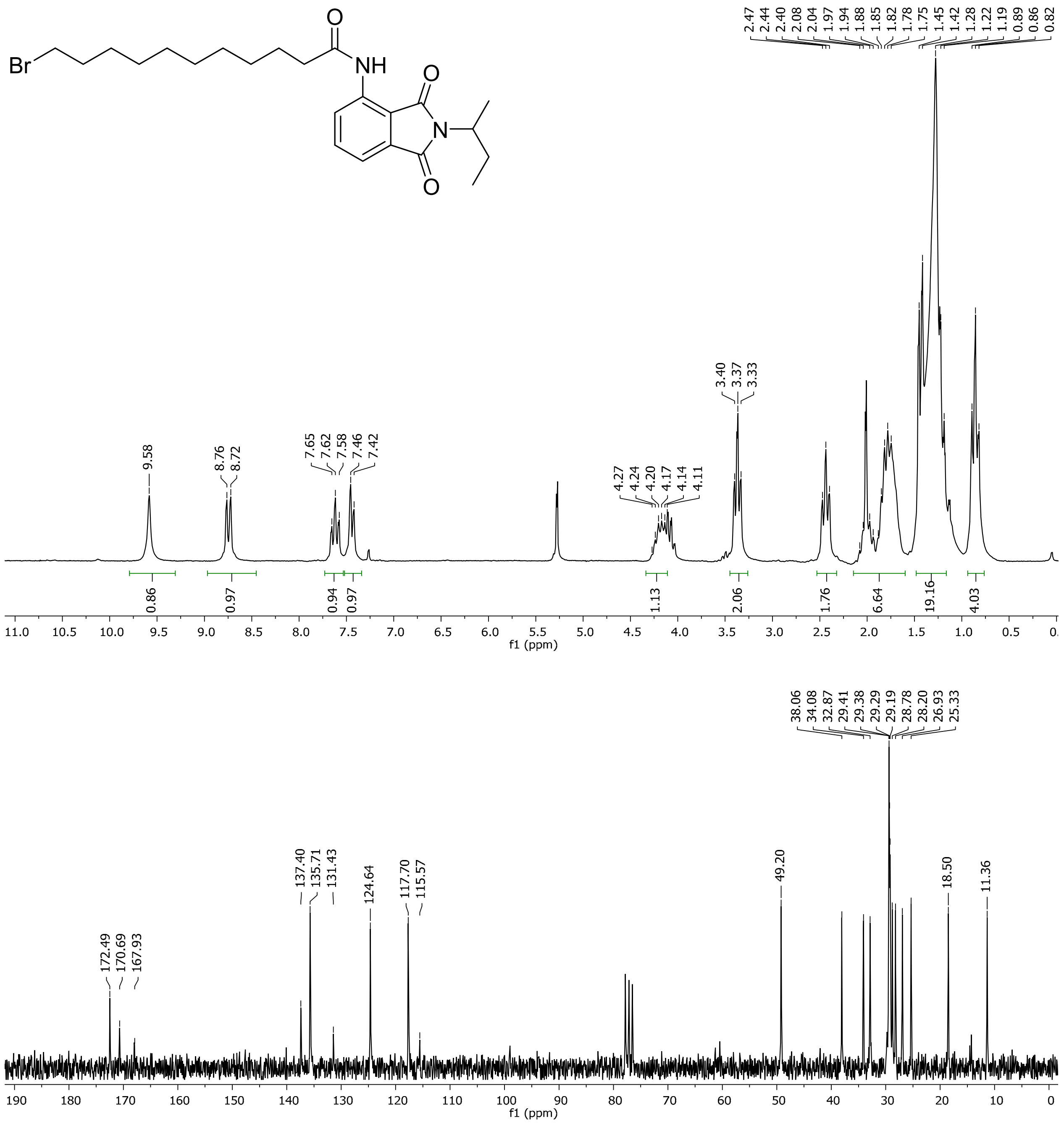

Figure S8. ^{1}H (200 MHz, top) and ^{13}C (50 MHz, bottom) NMR ($CDCl_3$) spectra of **8b**.

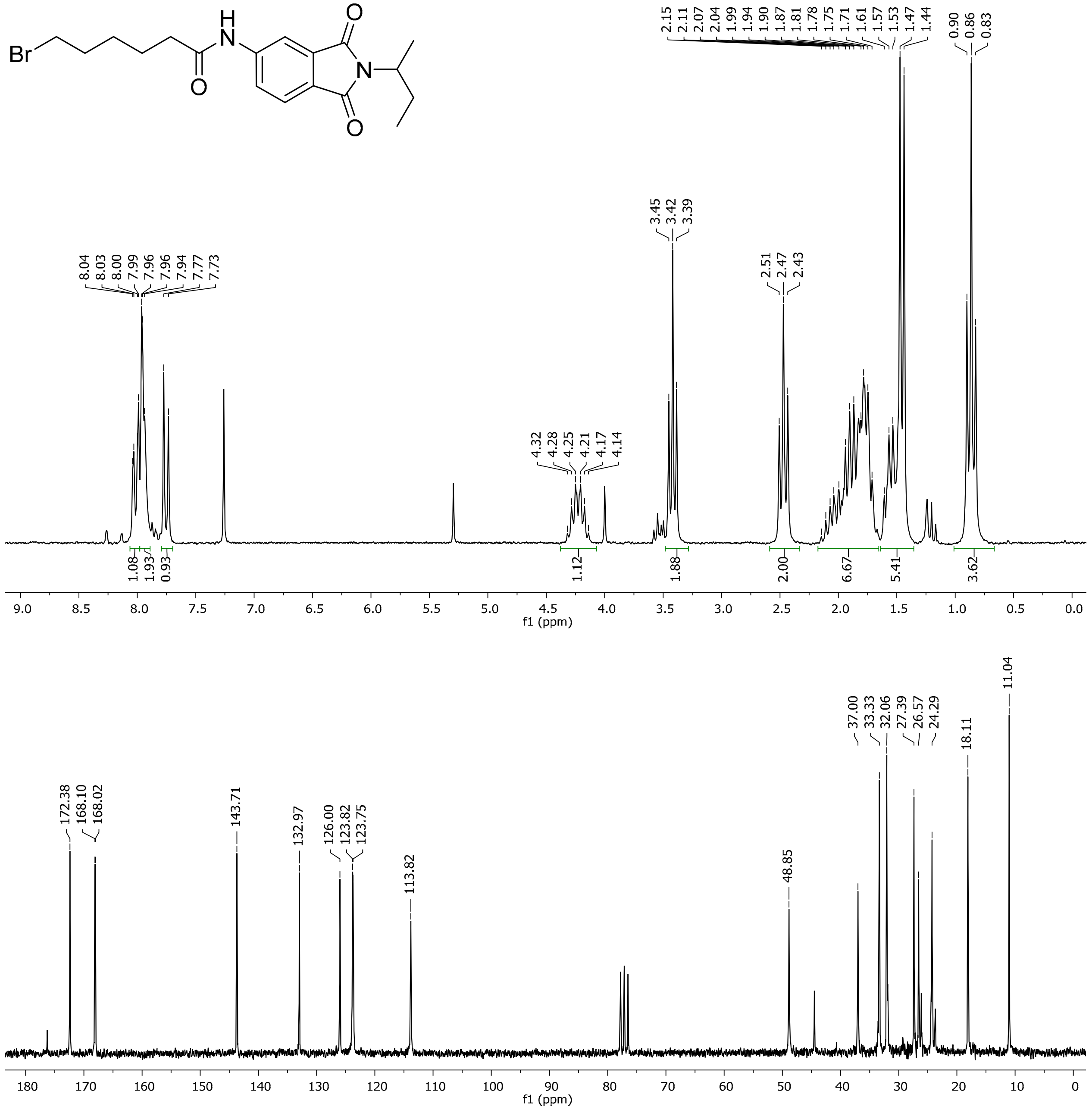

Figure S9. ^{1}H (200 MHz, top) and ^{13}C (50 MHz, bottom) NMR ($CDCl_3$) spectra of **9a** (mixture with 10mol% of the corresponding chloride).

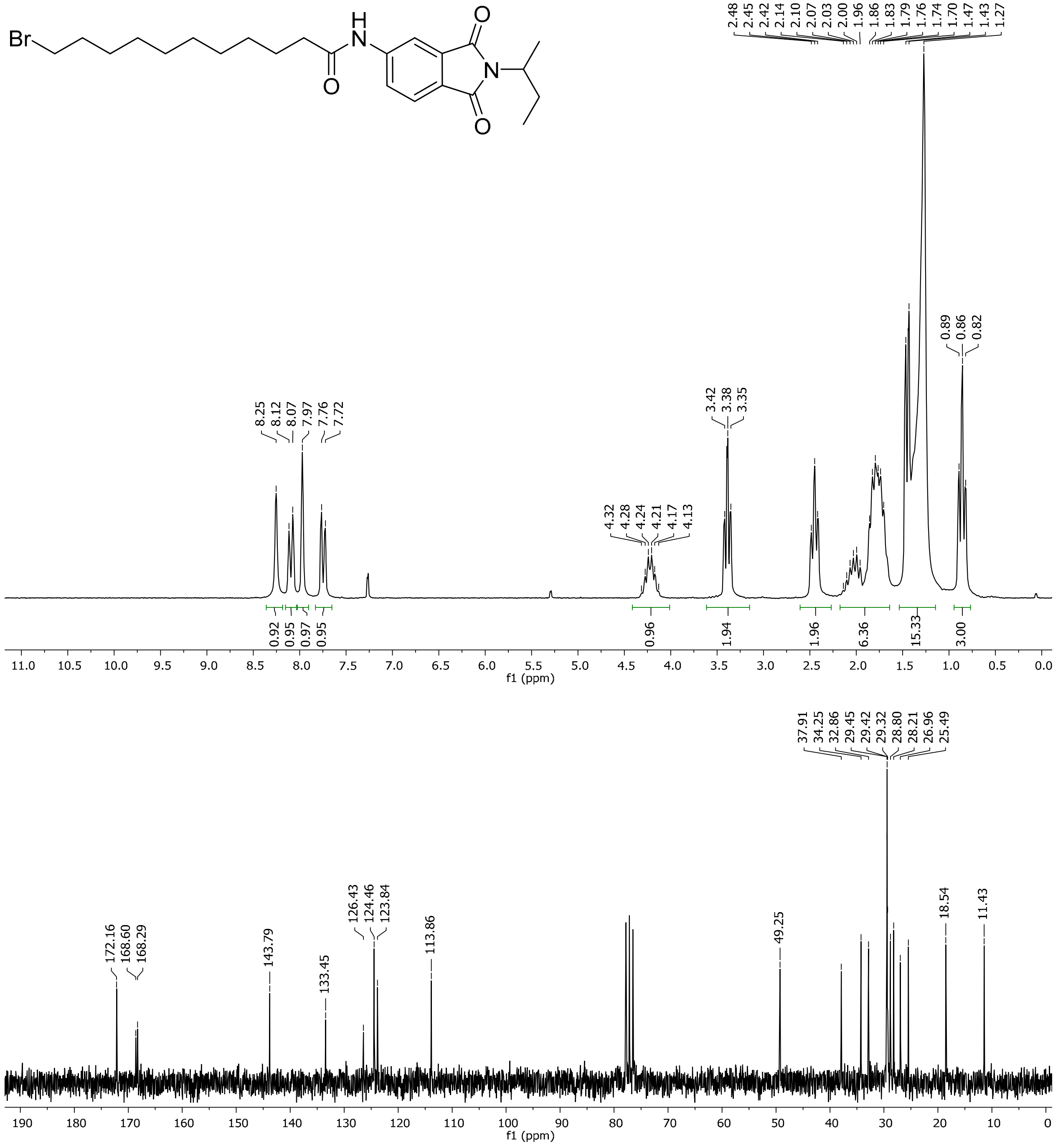

Figure S10. ^{1}H (200 MHz, top) and ^{13}C (50 MHz, bottom) NMR ($CDCl_3$) spectra of **9b**.

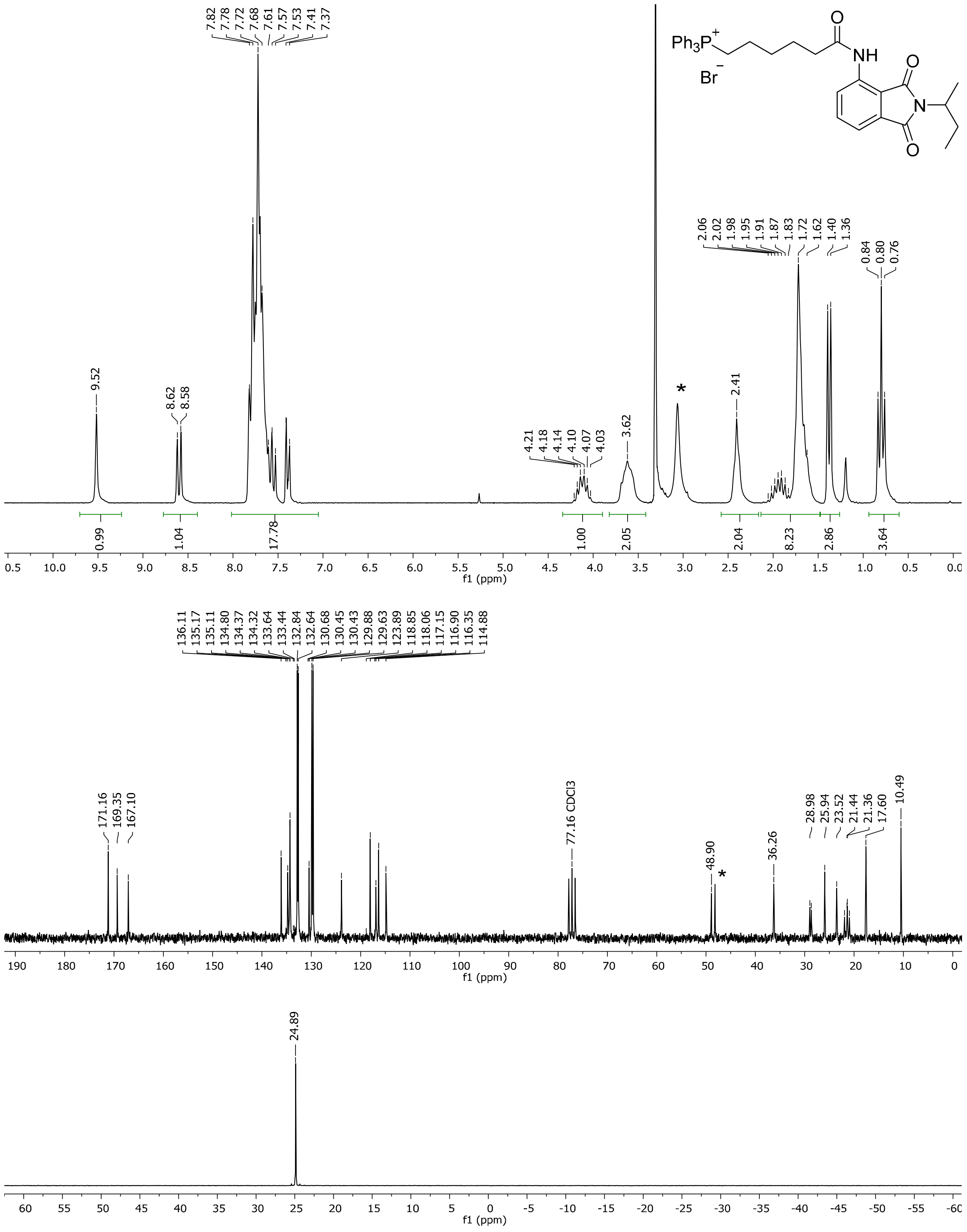

Figure S11. ^{1}H (200 MHz, top), ^{13}C (50 MHz, middle) and ^{31}P (81 MHz, bottom) NMR ($CDCl_3$) spectra of **10a**.

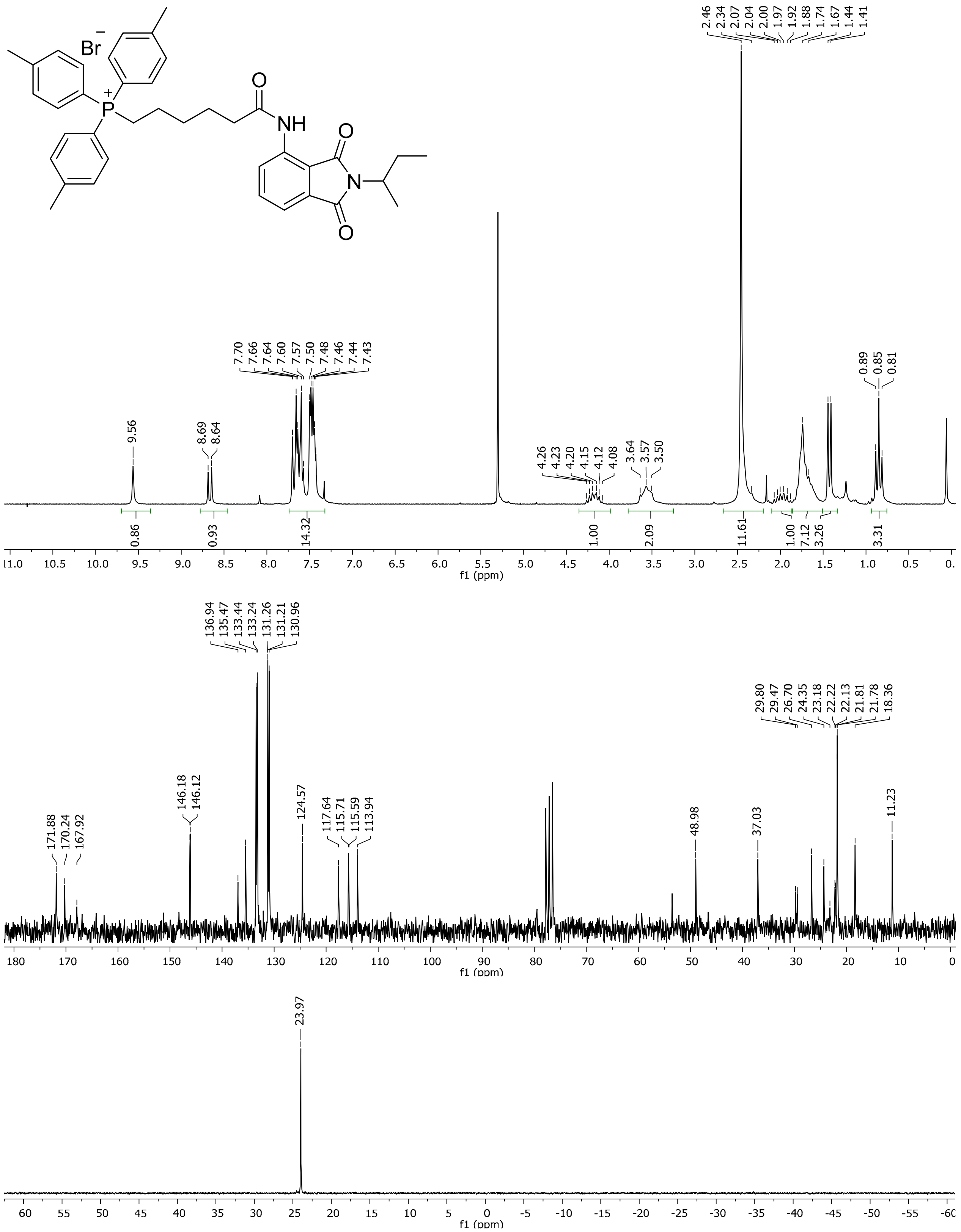

Figure S12. ^{1}H (200 MHz, top), ^{13}C (50 MHz, middle) and ^{31}P (81 MHz, bottom) NMR ($CDCl_3$) spectra of **10b**.

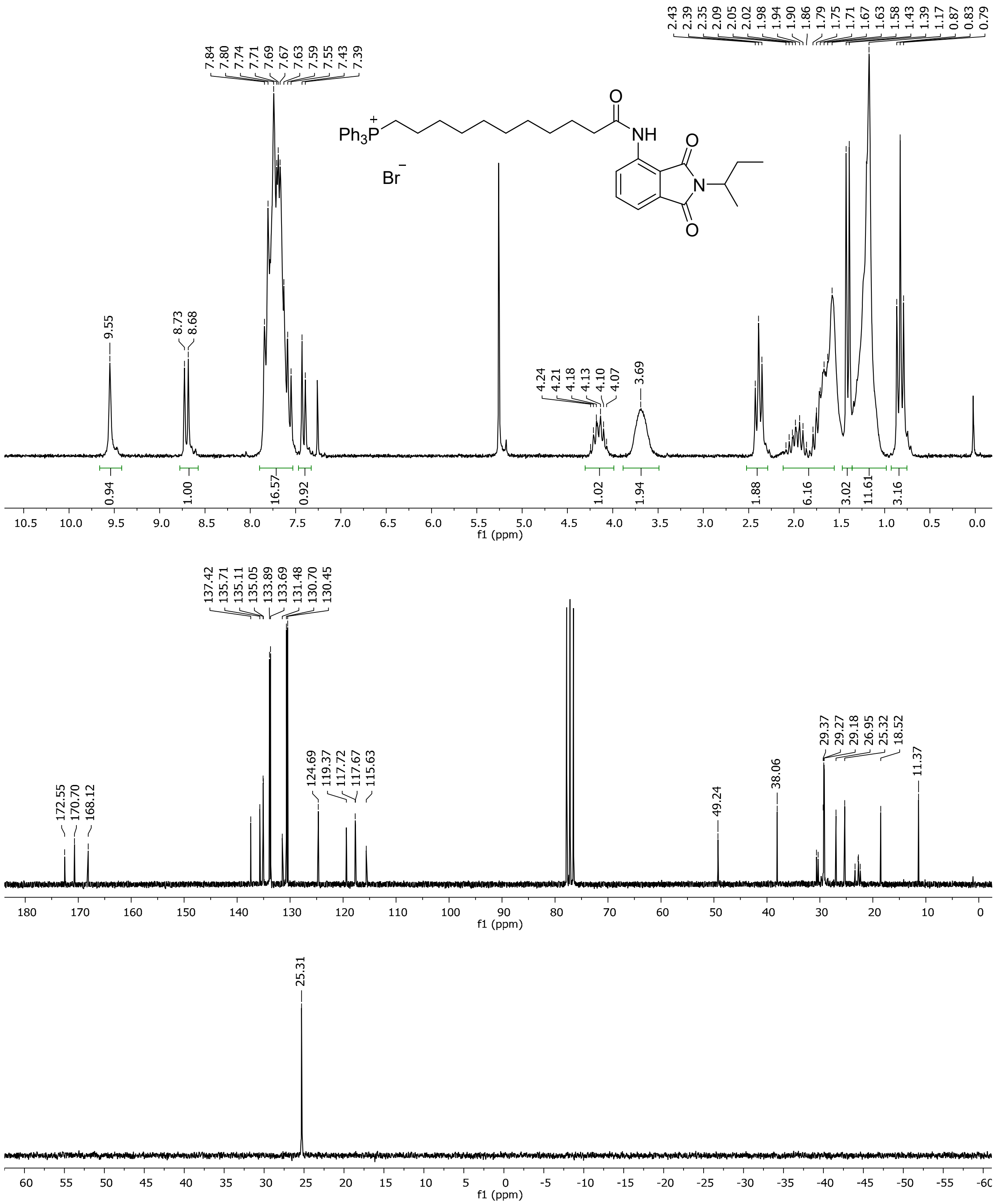

Figure S13. ^{1}H (200 MHz, top), ^{13}C (50 MHz, middle) and ^{31}P (81 MHz, bottom) NMR ($CDCl_3$) spectra of **10c**.

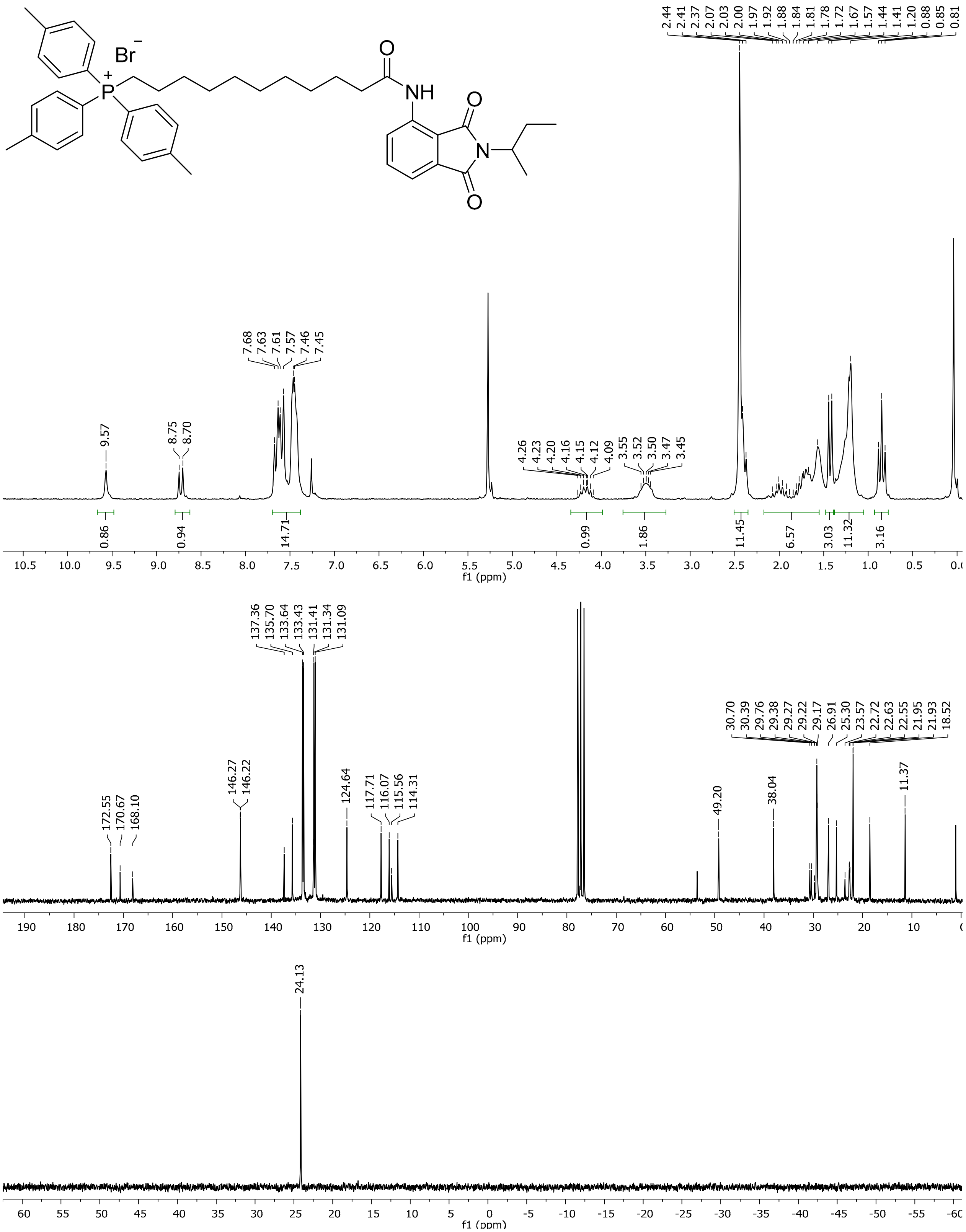

Figure S14. ^{1}H (200 MHz, top), ^{13}C (50 MHz, middle) and ^{31}P (81 MHz, bottom) NMR ($CDCl_3$) spectra of **10d**.

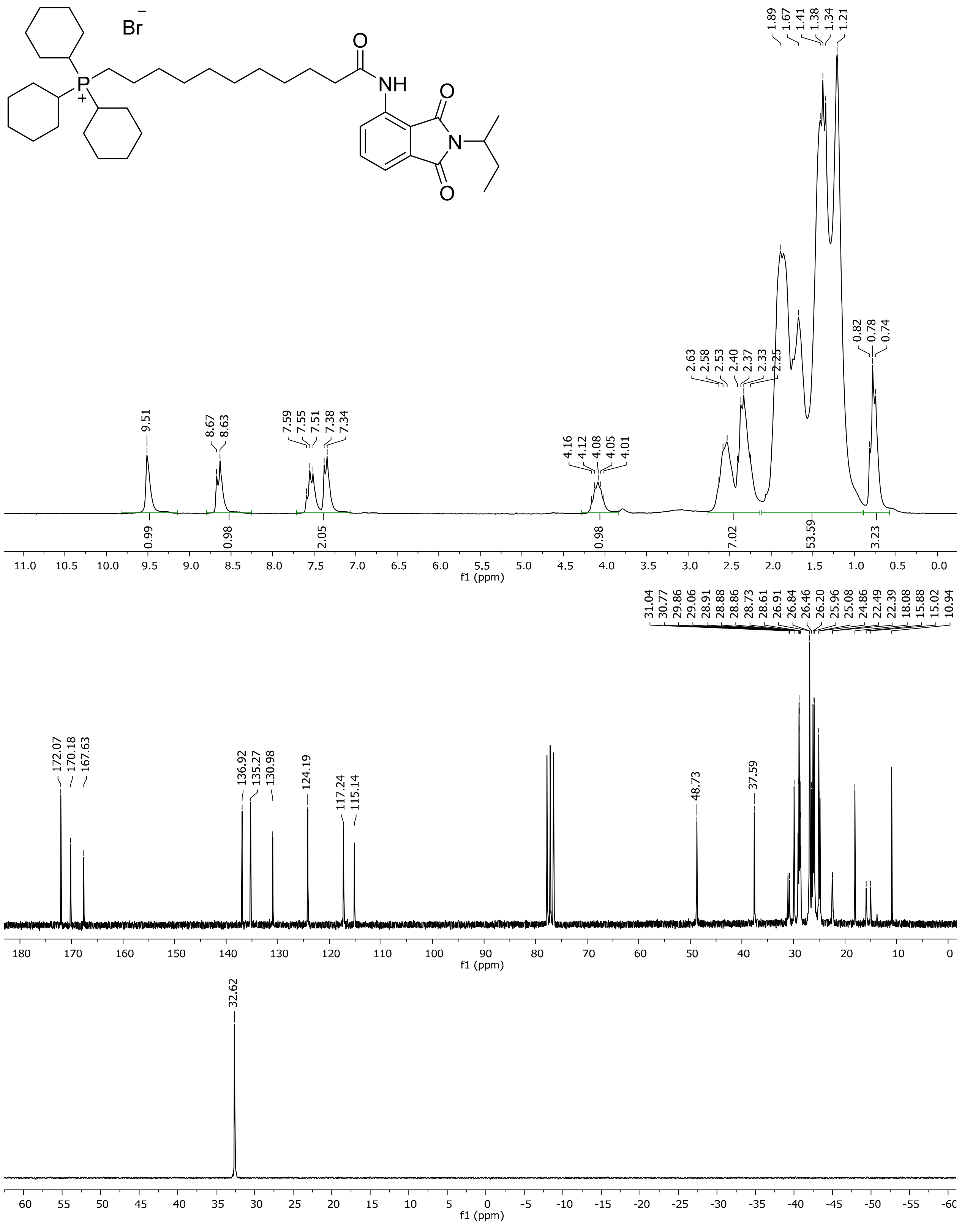

Figure S15. ^{1}H (200 MHz, top), ^{13}C (50 MHz, middle) and ^{31}P (81 MHz, bottom) NMR ($CDCl_3$) spectra of **10e**.

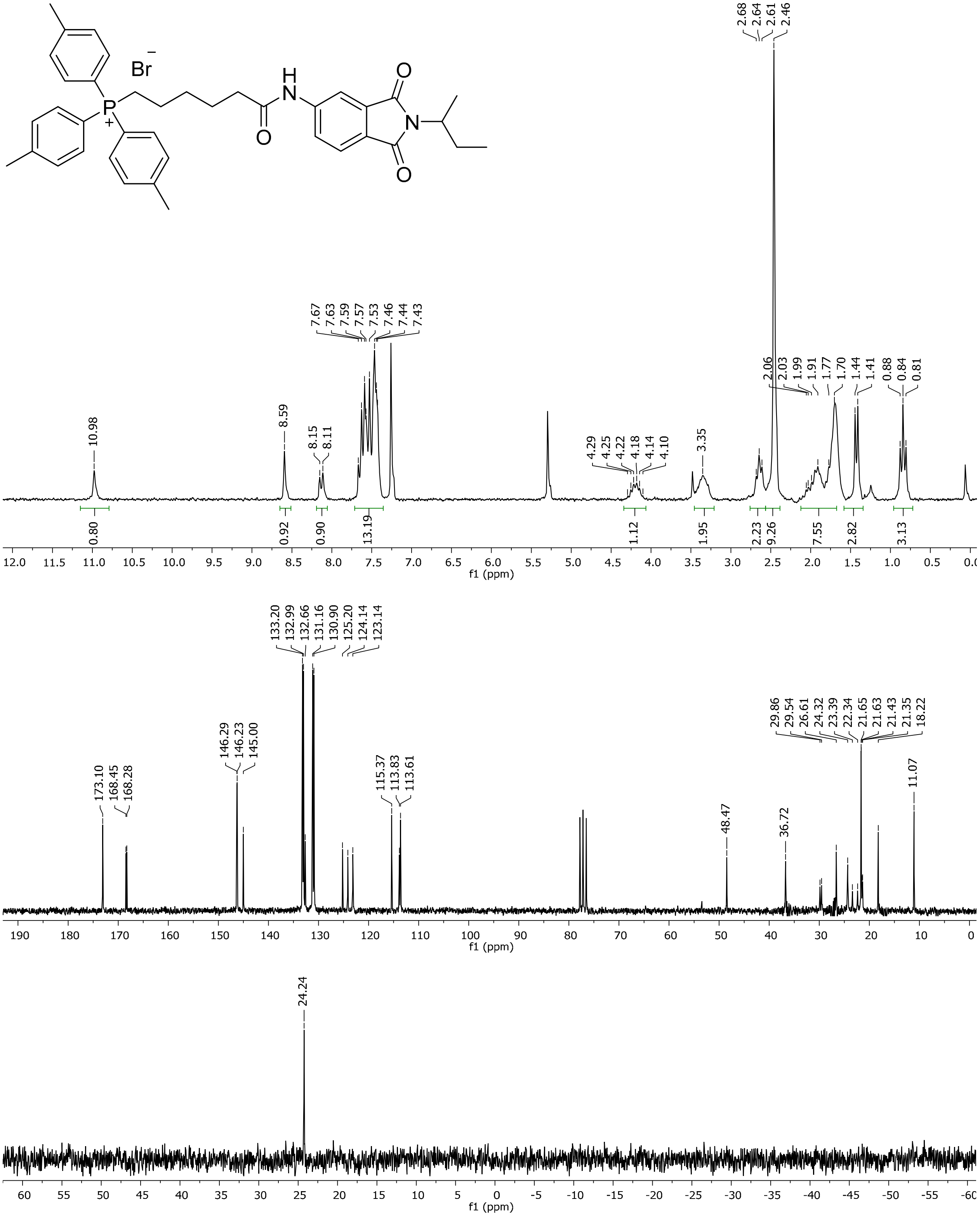

Figure S16. ^{1}H (200 MHz, top), ^{13}C (50 MHz, middle) and ^{31}P (81 MHz, bottom) NMR ($CDCl_3$) spectra of **11a**.

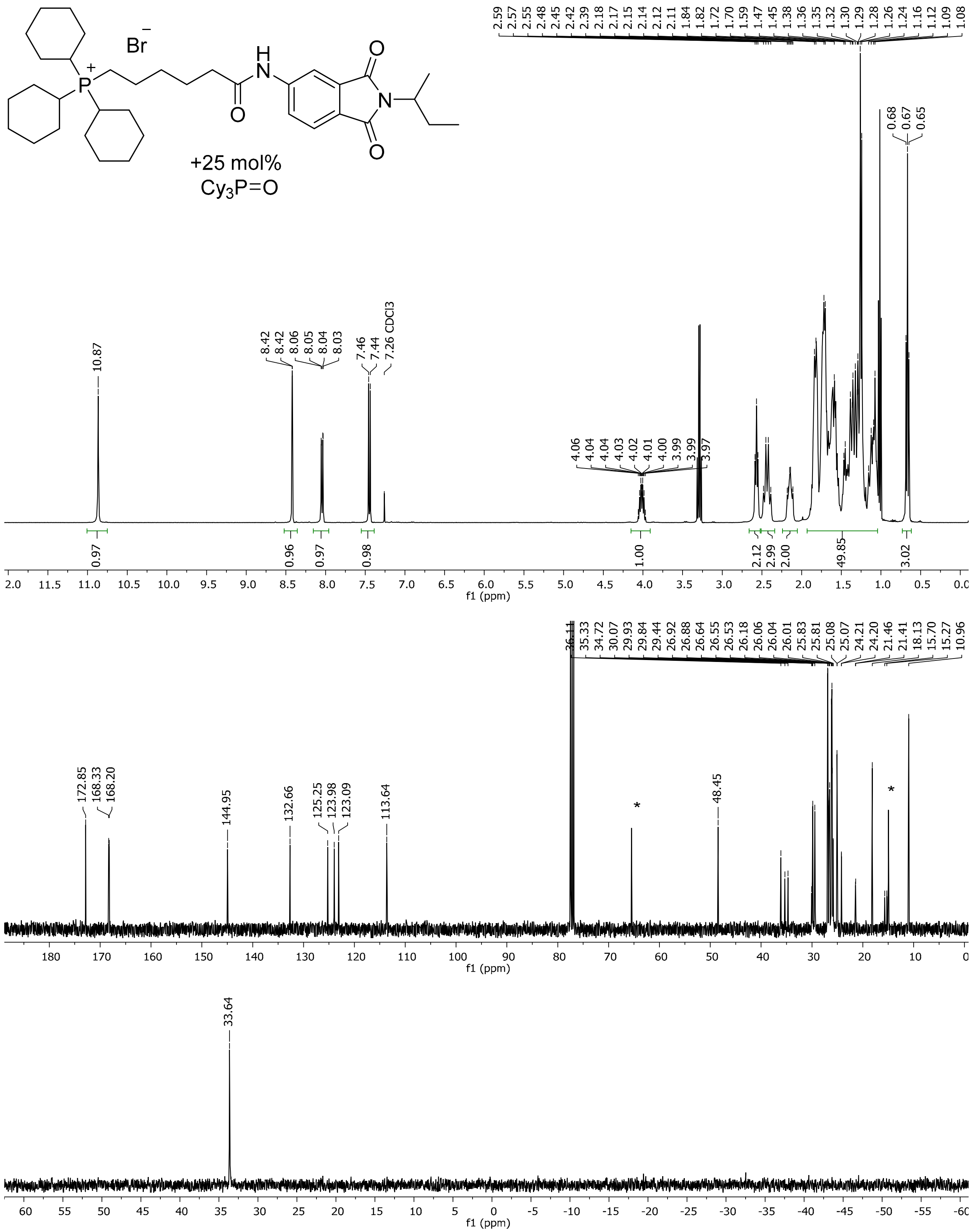

Figure S17. ^{1}H (400 MHz, top), ^{13}C (100 MHz, middle) and ^{31}P (81 MHz, bottom) NMR ($CDCl_3$) spectra of **11b** (contaminated with 25mol% $Cy_3P{=}O$).

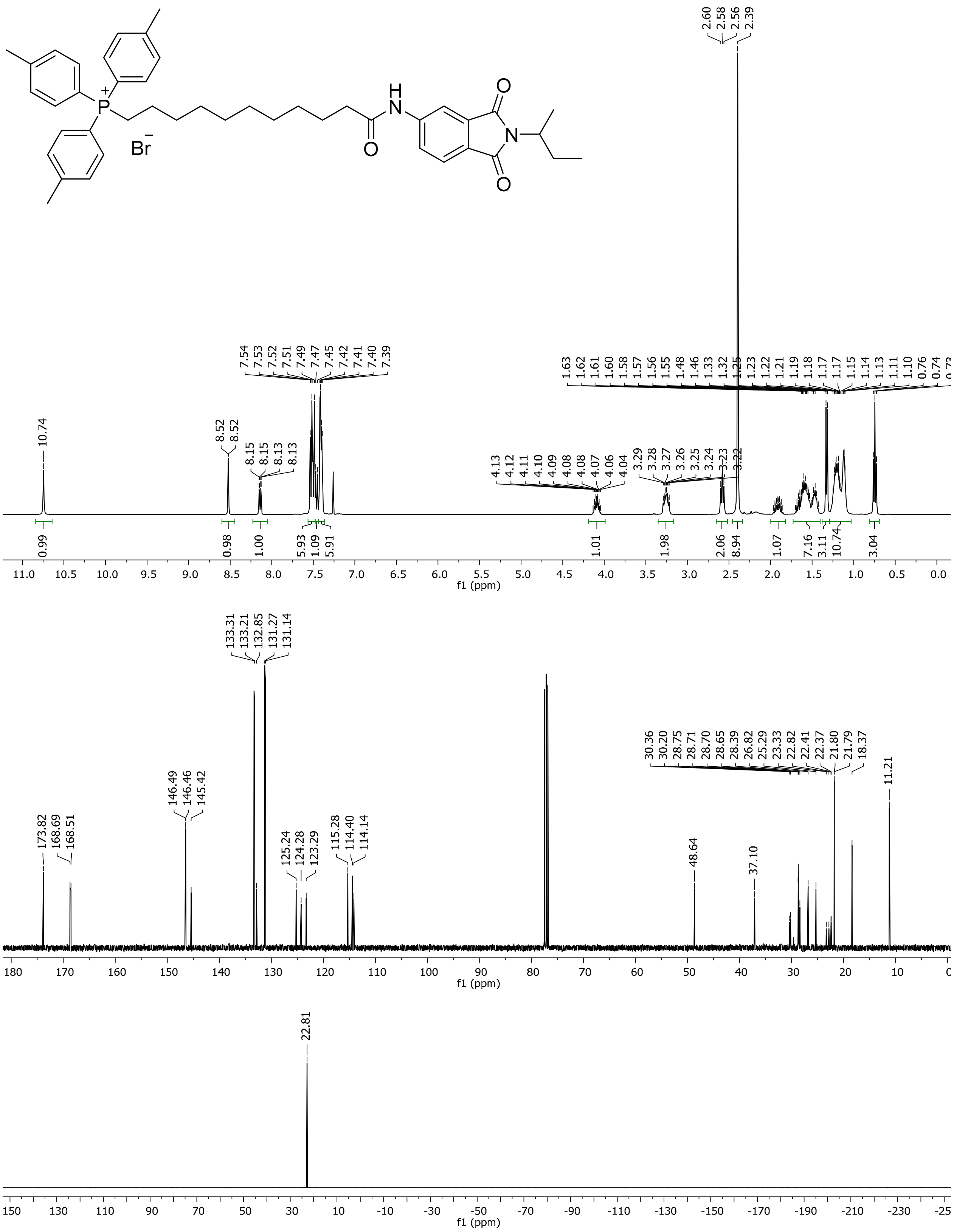

Figure S18. ^{1}H (400 MHz, top), ^{13}C (100 MHz, middle) and ^{31}P (81 MHz, bottom) NMR ($CDCl_3$) spectra of **11c**.

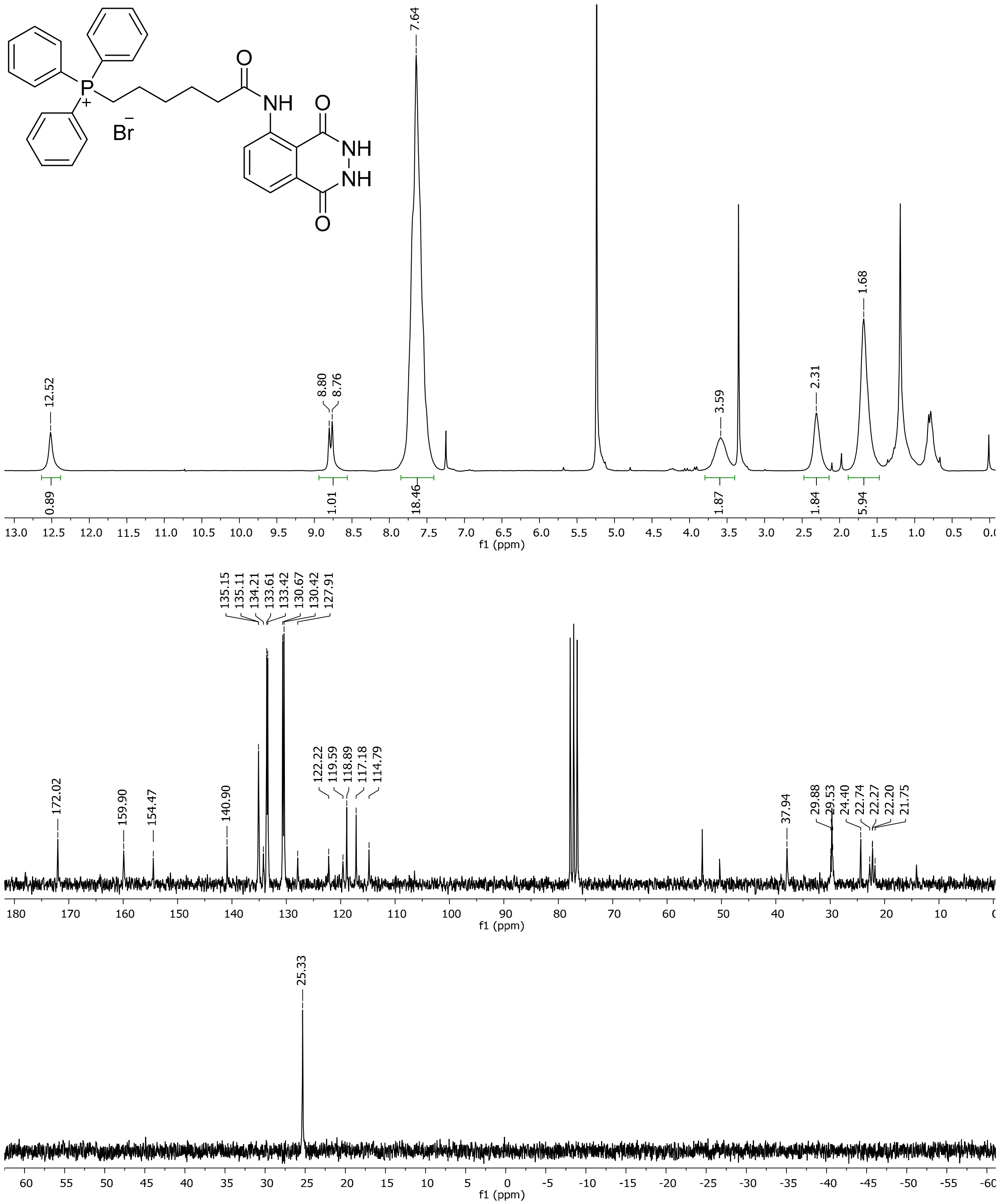

Figure S19. ^{1}H (200 MHz, top), ^{13}C (50 MHz, middle) and ^{31}P (81 MHz, bottom) NMR ($CDCl_3$) spectra of **1a**.

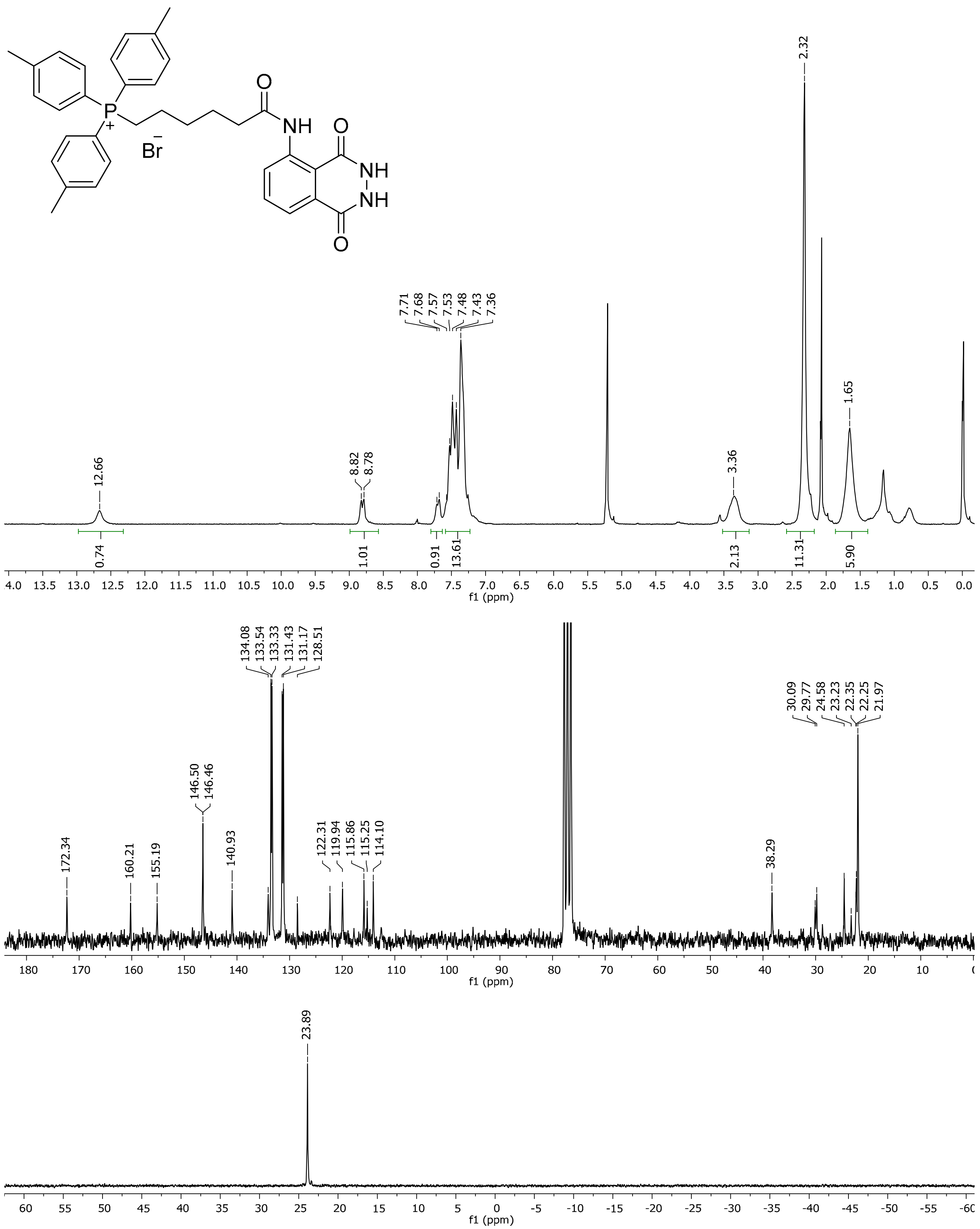

Figure S20. ^{1}H (200 MHz, top), ^{13}C (50 MHz, middle) and ^{31}P (81 MHz, bottom) NMR ($CDCl_3$) spectra of **1b**.

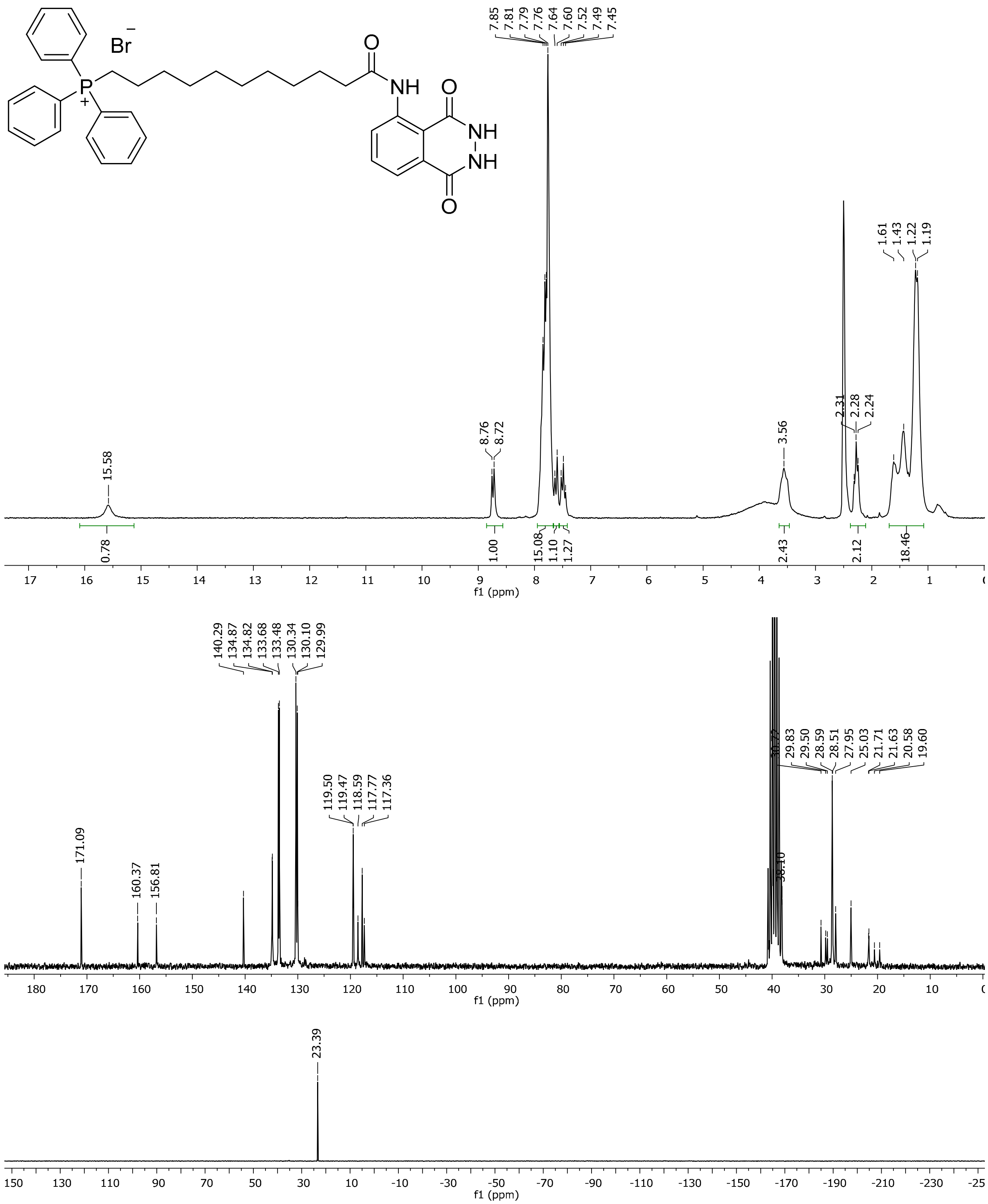

Figure S21. ^{1}H (200 MHz, top), ^{13}C (50 MHz, middle) and ^{31}P (81 MHz, bottom) NMR (DMSO-d_6) spectra of **1c**.

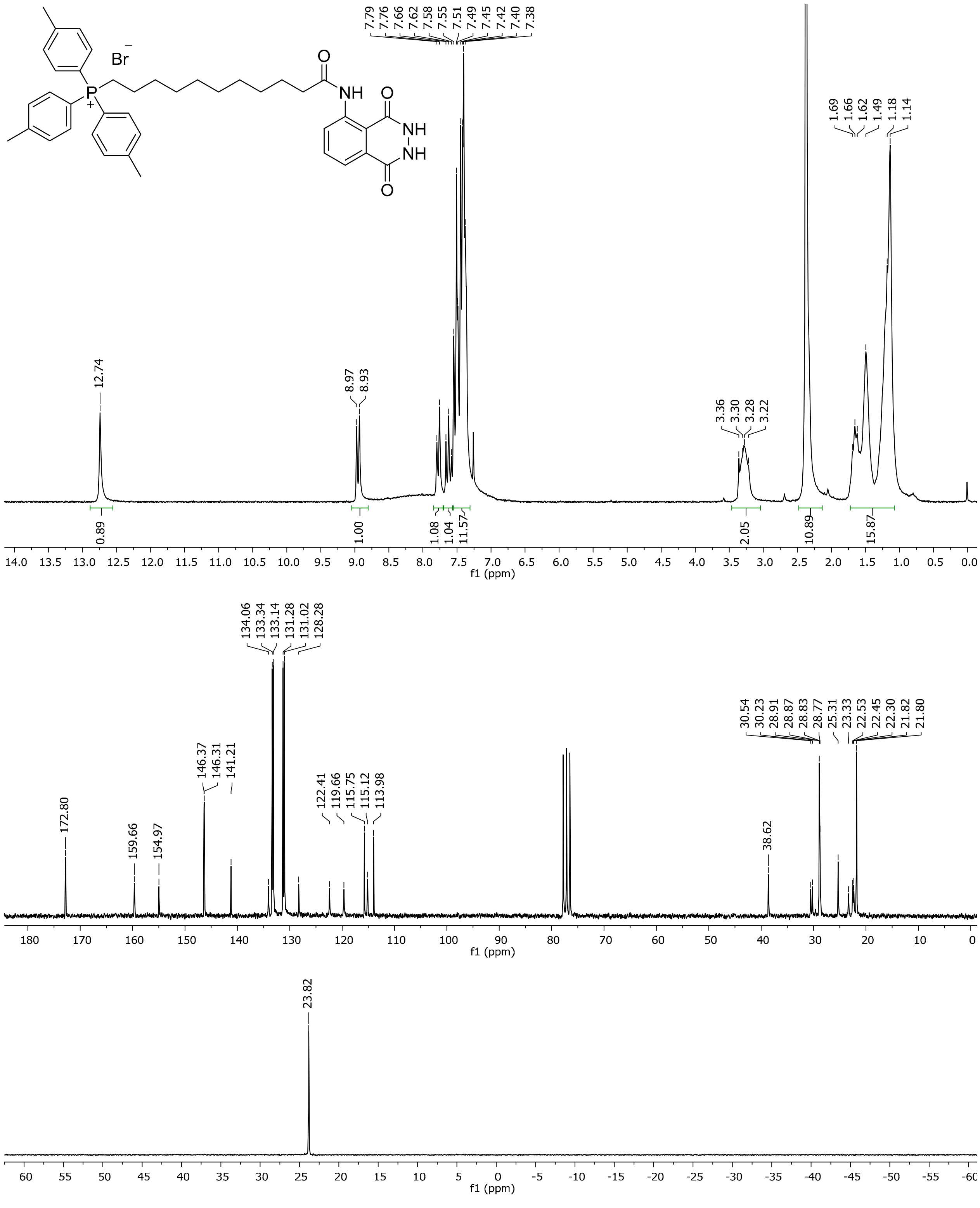

Figure S22. ^{1}H (200 MHz, top), ^{13}C (50 MHz, middle) and ^{31}P (81 MHz, bottom) NMR ($CDCl_3$) spectra of **1d**.

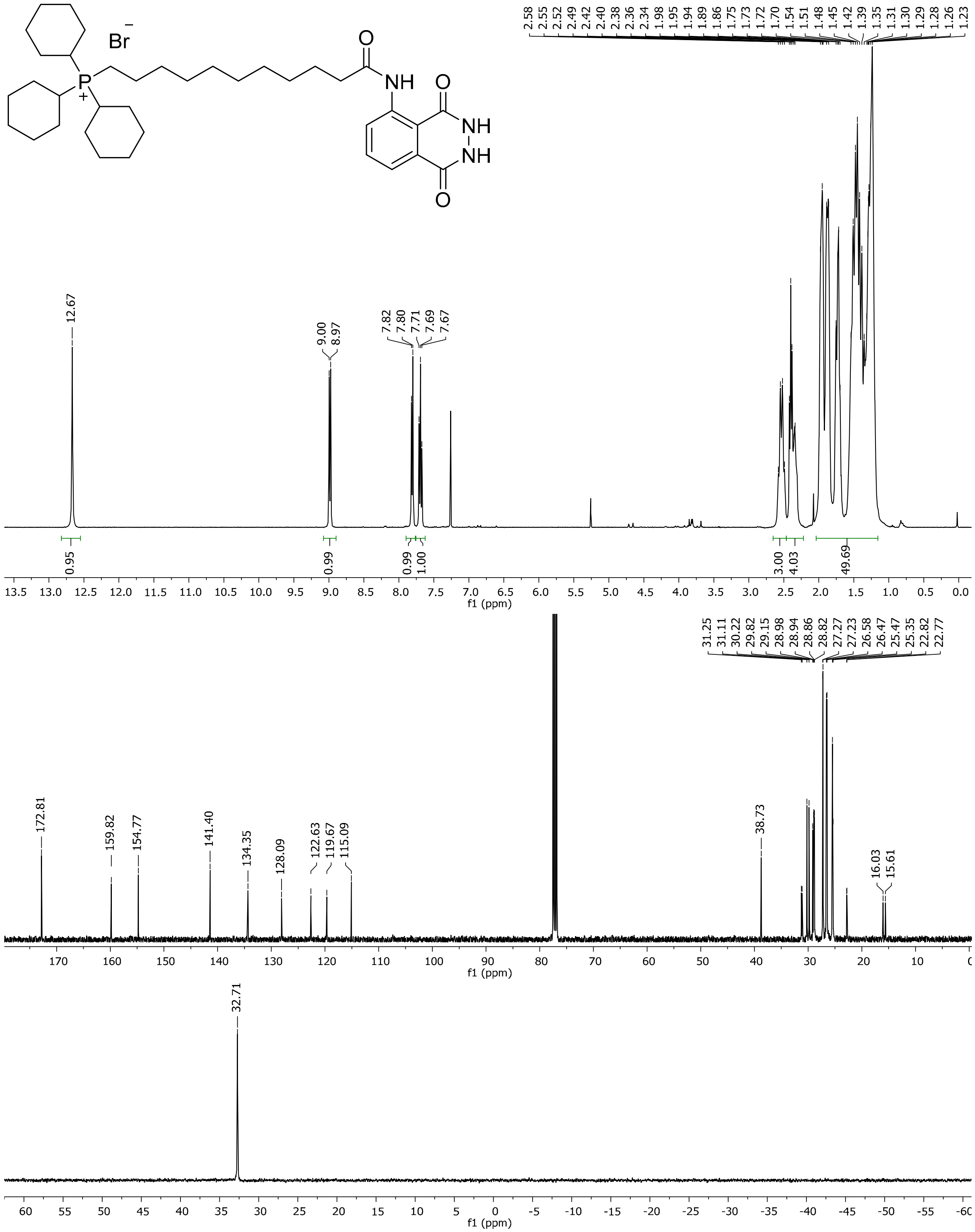

Figure S23. ^{1}H (400 MHz, top), ^{13}C (100 MHz, middle) and ^{31}P (81 MHz, bottom) NMR ($CDCl_3$) spectra of **1e**.

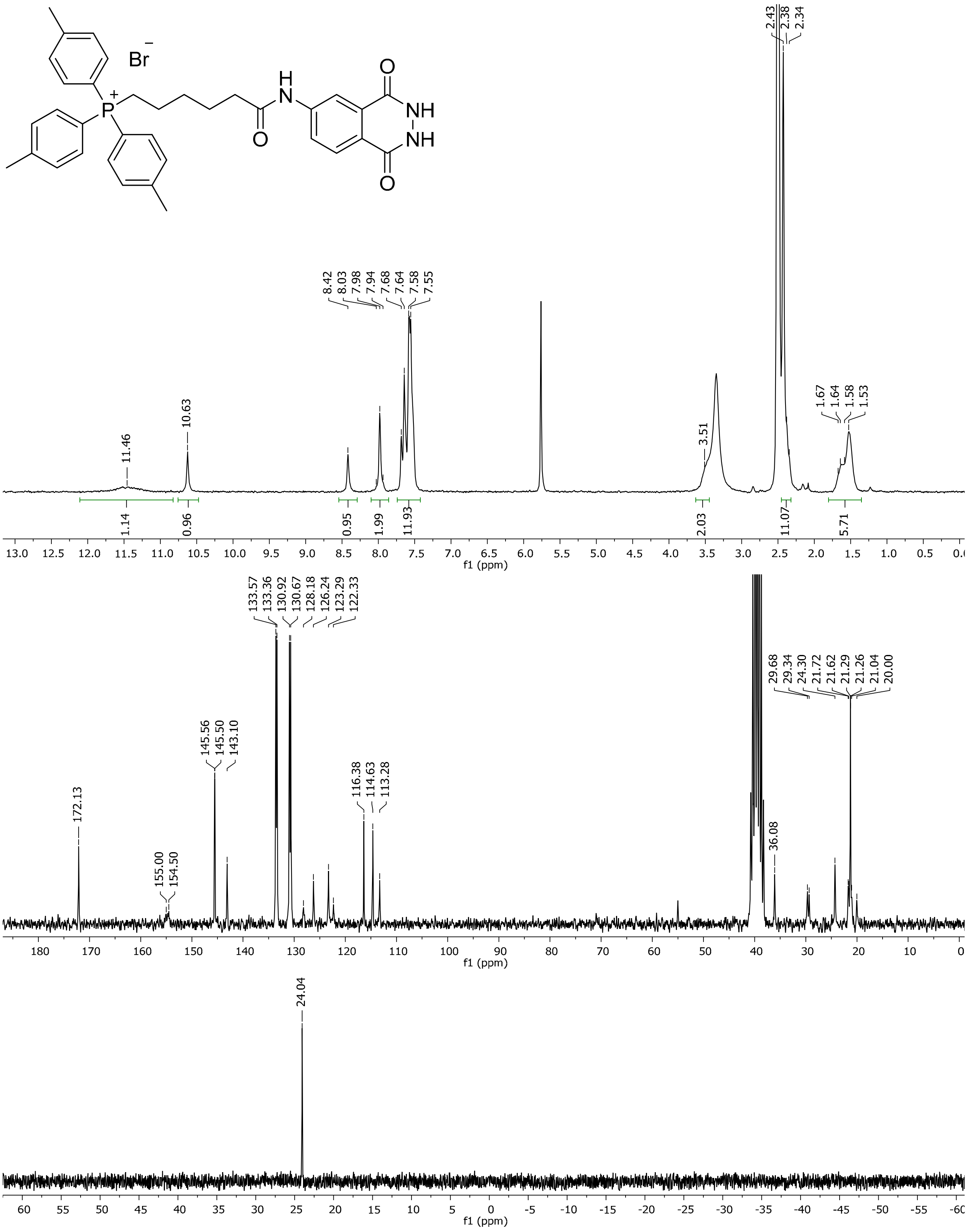

Figure S24. ^{1}H (200 MHz, top), ^{13}C (50 MHz, middle) and ^{31}P (81 MHz, bottom) NMR (DMSO-d_6) spectra of **12a**.

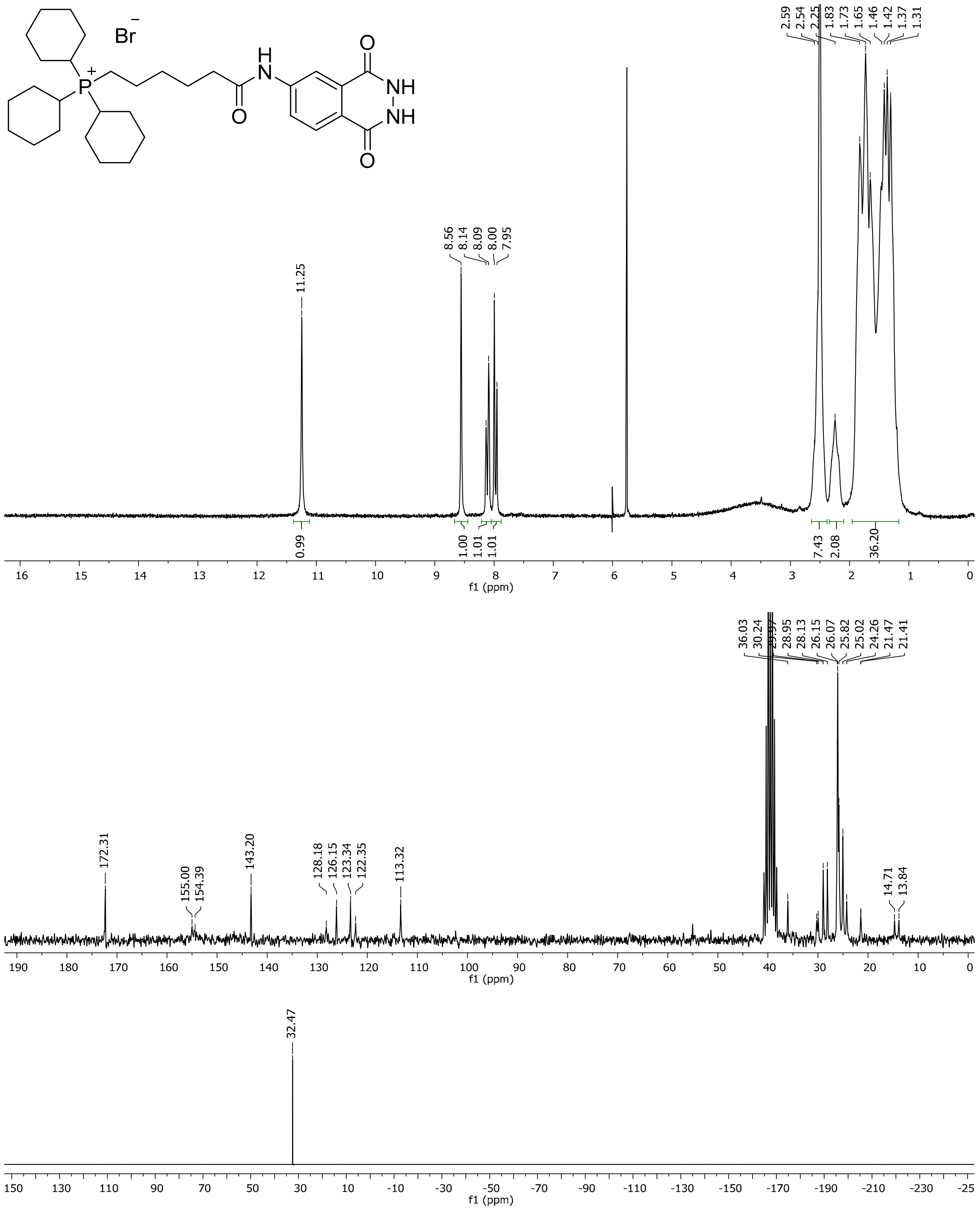

Figure S25. ^{1}H (200 MHz, top), ^{13}C (50 MHz, middle) and ^{31}P (162 MHz, bottom) NMR (DMSO-d_6) spectra of **12b**.

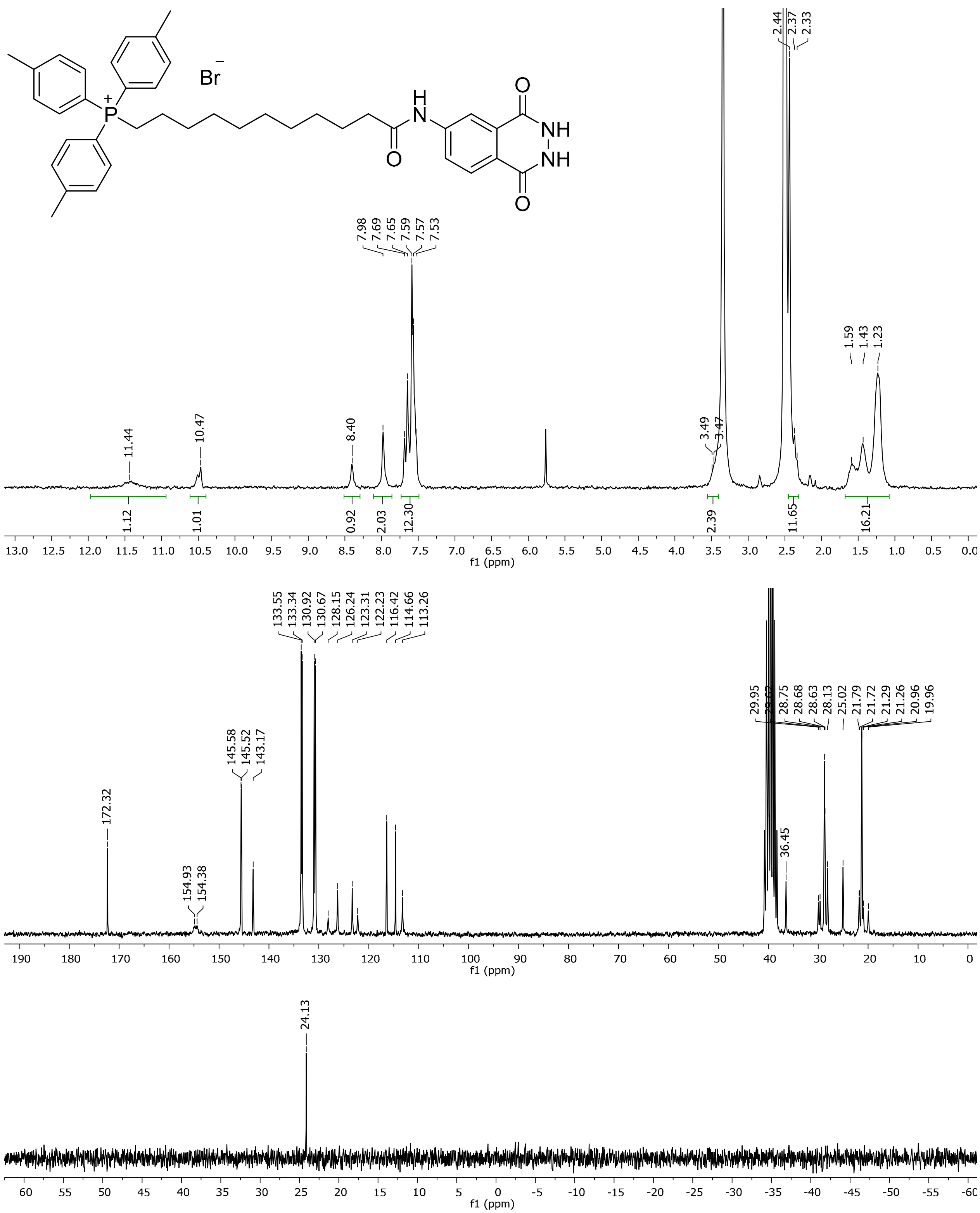

Figure S26. ^{1}H (200 MHz, top), ^{13}C (50 MHz, middle) and ^{31}P (81 MHz, bottom) NMR (DMSO-d_6) spectra of **12c**.

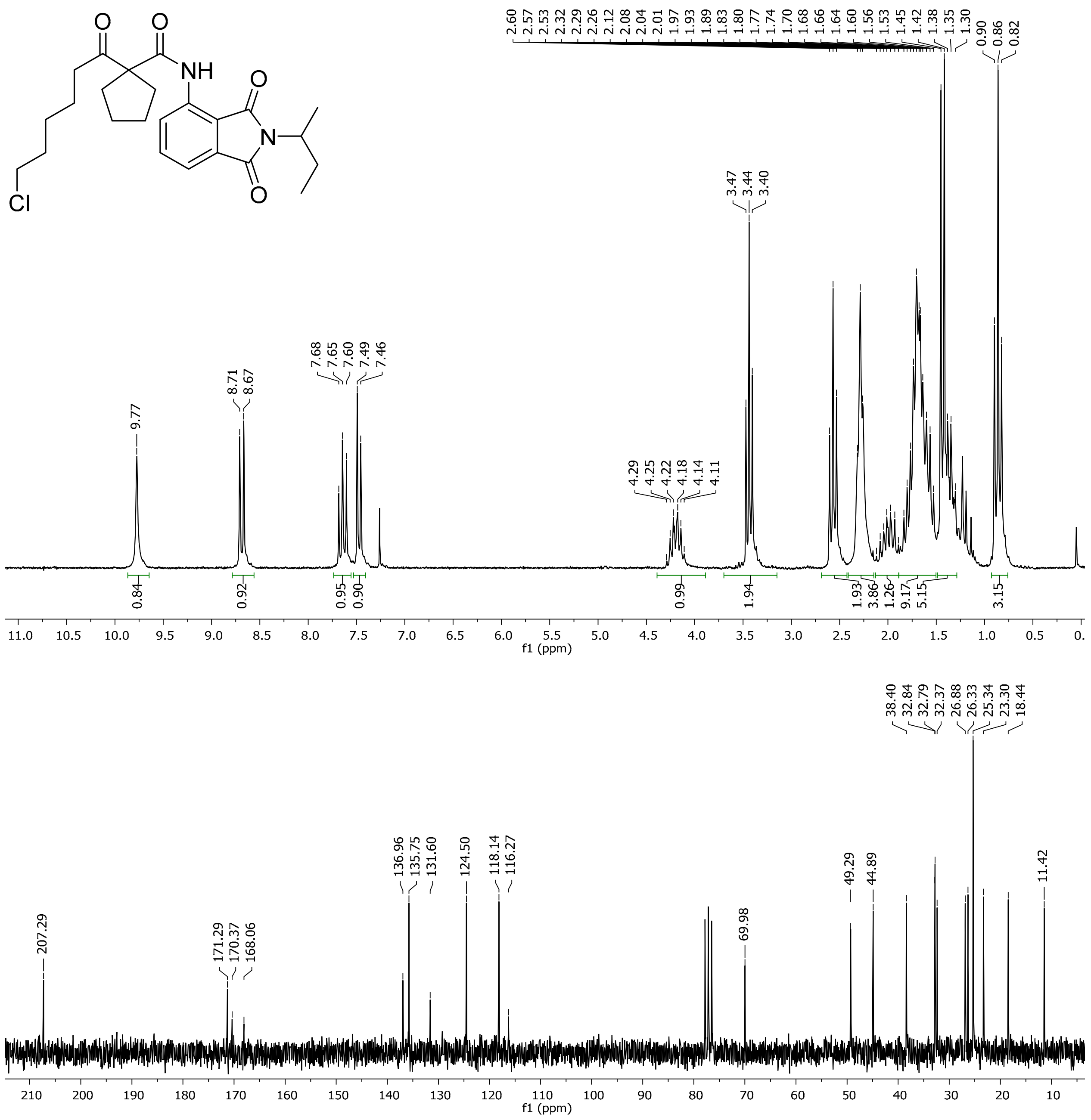

Figure S27. ^{1}H (200 MHz, top) and ^{13}C (50 MHz, bottom) NMR ($CDCl_3$) spectra of **13**.

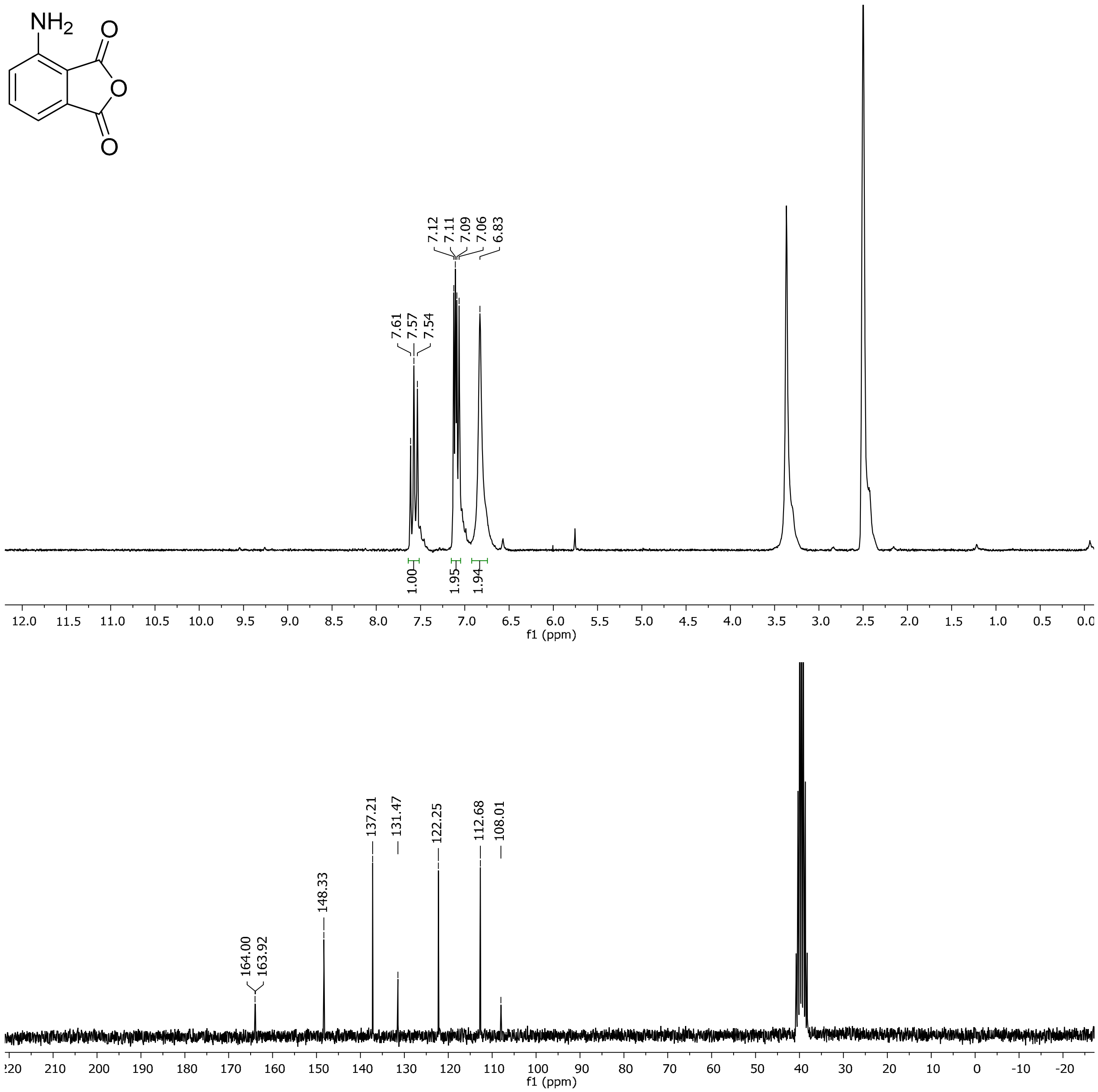

Figure S28. ^{1}H (200 MHz, top) and ^{13}C (50 MHz, bottom) NMR (DMSO-d_6) spectra of **15**.

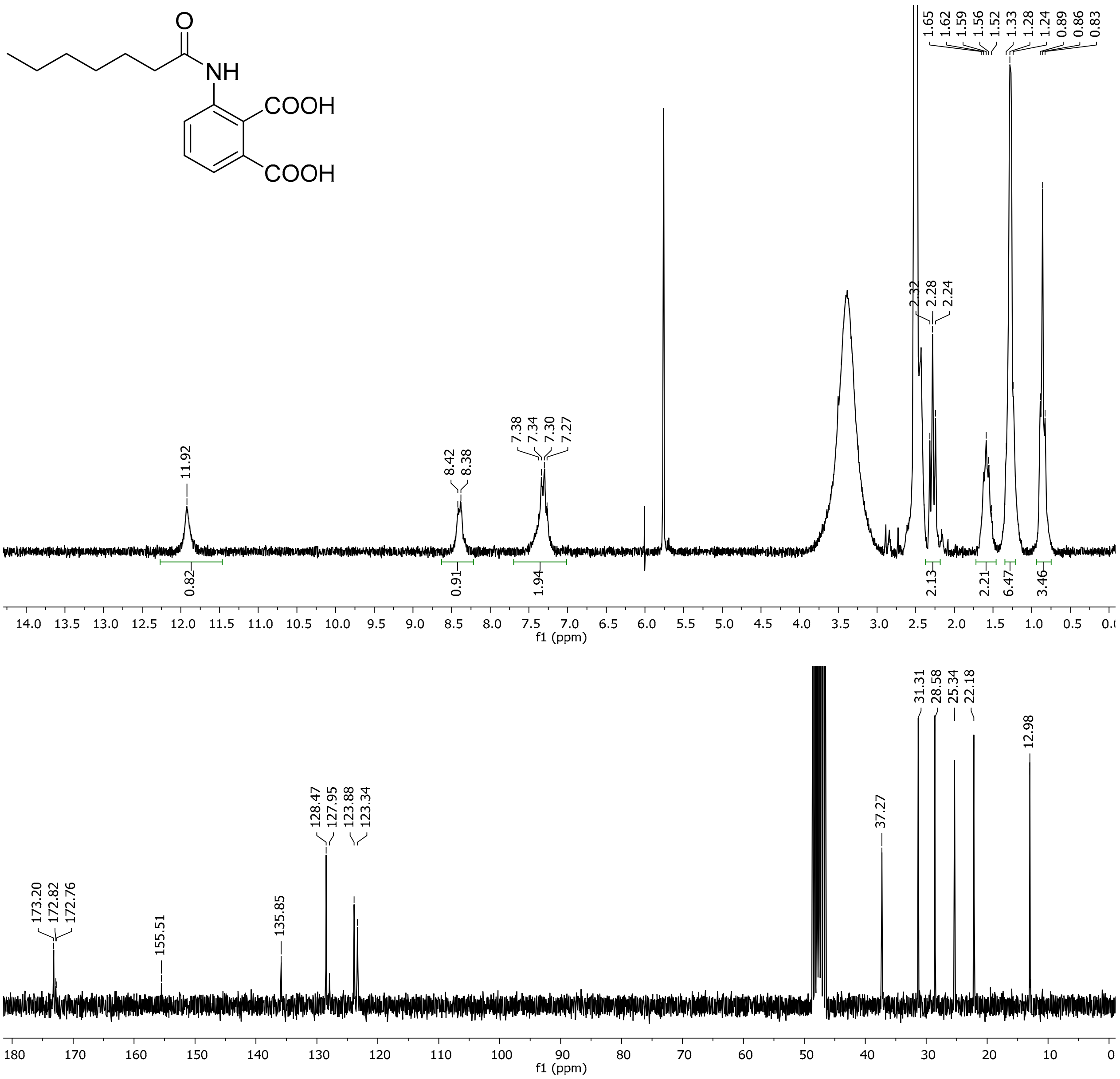

Figure S29. ^{1}H (200 MHz, top) and ^{13}C (63 MHz, bottom) NMR (MeOD-d_4) spectra of **14**.
